# Towards more accurate microbial source tracking via non-negative matrix factorization (NMF)

**DOI:** 10.1093/bioinformatics/btae227

**Published:** 2024-06-28

**Authors:** Ziyi Huang, Dehan Cai, Yanni Sun

**Affiliations:** Department of Electrical Engineering, City University of Hong Kong, Kowloon, Hong Kong SAR, China; Department of Electrical Engineering, City University of Hong Kong, Kowloon, Hong Kong SAR, China; Department of Electrical Engineering, City University of Hong Kong, Kowloon, Hong Kong SAR, China

## Abstract

**Motivation:**

The microbiome of a sampled habitat often consists of microbial communities from various sources, including potential contaminants. Microbial source tracking (MST) can be used to discern the contribution of each source to the observed microbiome data, thus enabling the identification and tracking of microbial communities within a sample. Therefore, MST has various applications, from monitoring microbial contamination in clinical labs to tracing the source of pollution in environmental samples. Despite promising results in MST development, there is still room for improvement, particularly for applications where precise quantification of each source’s contribution is critical.

**Results:**

In this study, we introduce a novel tool called SourceID-NMF towards more precise microbial source tracking. SourceID-NMF utilizes a non-negative matrix factorization (NMF) algorithm to trace the microbial sources contributing to a target sample. By leveraging the taxa abundance in both available sources and the target sample, SourceID-NMF estimates the proportion of available sources present in the target sample. To evaluate the performance of SourceID-NMF, we conducted a series of benchmarking experiments using simulated and real data. The simulated experiments mimic realistic yet challenging scenarios for identifying highly similar sources, irrelevant sources, unknown sources, low abundance sources, and noise sources. The results demonstrate the superior accuracy of SourceID-NMF over existing methods. Particularly, SourceID-NMF accurately estimated the proportion of irrelevant and unknown sources while other tools either over- or under-estimated them. In addition, the noise sources experiment also demonstrated the robustness of SourceID-NMF for MST.

**Availability and implementation:**

SourceID-NMF is available online at https://github.com/ZiyiHuang0708/SourceID-NMF.

## 1 Introduction

Microorganisms, often referred to as microbes, are integral to the functioning of various ecosystems (Qin *et al.* 2010, Philippot *et al.* 2013, Belkaid and Hand 2014). The microbial community refers to the sum total of microbes that interact in a specific area, such as the soil microbiome, skin microbiome, gut microbiome, etc. Because the microbiome is dynamic and can be affected by various factors such as location, environmental conditions, and interactions with other organisms (David *et al.* 2014), different microbial communities can exhibit very distinct compositions, even if they belong to the same type of ecosystem (Fierer and Jackson 2006). In addition, the interaction between microbial communities also promotes changes in microbial communities (Faust and Raes 2012).

To better comprehend the changes of microbial communities, it is essential to identify their origins and pathways. This necessity has given rise to the field of microbial source tracking (MST), which assumes that a “sink” microbial community is a mixture of multiple “source” microbial communities and aims to determine the contribution of each source to the sink (Simpson *et al.* 2002, Knights *et al.* 2011). MST has a wide range of applications. Initially, it was primarily used for monitoring the sources of microbial contamination in environmental samples, particularly in water (Simpson *et al.* 2002, Wu *et al.* 2010). Furthermore, MST helps eliminate unexpected microbiomes originating from irrelevant sources in the sample, such as the microbiomes associated with laboratory contamination (Zhu *et al.* 2020). In addition, tracking the interaction of microbial communities in intensive care units (ICUs) provides insights into care-taking for patients and newborns (Hewitt *et al.* 2013, Brooks *et al.* 2014).

Several methods have been proposed for tracking microbial sources. Early source tracking approaches have been employed to monitor water contamination (Simpson *et al.* 2002, Greenberg *et al.* 2010, Smith *et al.* 2010, Wu *et al.* 2010). Some methods (Simpson *et al.* 2002, Wu *et al.* 2010) only rely on checking specific species (e.g. species that are commonly found in fecal sources), which may introduce bias in the tracking process. On the other hand, more advanced methods (Greenberg *et al.* 2010, Smith *et al.* 2010) aim to predict contaminating sources using all isolates (species) within the entire community. However, the prediction in these methods relies on the antibiotic resistance profile of each species, thereby limiting contamination tracking to sources with antibiotic resistance information.

Metagenomic sequencing has become a powerful approach for investigating microbial communities because it can sequence all the genetic materials in a sample. As metagenomic data allows comprehensive microbial composition analysis, using all the derived taxa for MST is expected to generate higher accuracy. Numerous metagenomic datasets have been sequenced for microbiome in various ecosystems (Turnbaugh *et al.* 2007, Thompson *et al.* 2017). Correspondingly, computational tools have been developed to quantify the microbial composition, including taxa and their abundance, from metagenomic sequencing data (Caporaso *et al.* 2010, Bolyen *et al.* 2019, Wood *et al.* 2019). By using the derived taxonomic profiles, we can compare microbial compositions across different sources and track their overall contributions within a given sample.

Two widely used methods of MST, SourceTracker (Knights *et al.* 2011) and FEAST (Shenhav *et al.* 2019), have developed probabilistic frameworks for tracking microbial sources using all species in microbial samples. Both methods take the taxa abundance in reference sources and the target sample as input, providing estimated proportions of each reference source in the target sample. SourceTracker employs Gibbs sampling, which has a trade-off between speed and accuracy, while FEAST utilizes a maximum likelihood method for faster computation (Knights *et al.* 2011, Shenhav *et al.* 2019). Despite the promising results achieved by SourceTracker and FEAST, there is still room for improvement, especially in applications where precise quantification of each source’s contribution is of utmost importance.

In this study, we developed a tool called SourceID-NMF for tracking microbial sources. SourceID-NMF estimates the contributions of sources to a target sample by applying a non-negative matrix factorization (NMF) model to the observed taxa abundance in the sources and the target sample. We compared SourceID-NMF with state-of-the-art tools using simulated and real data. Simulated experiments demonstrated that SourceID-NMF outperformed other tools under different scenarios. Specifically, SourceID-NMF accurately estimated proportions of unknown sources and proved to be well-suited in scenarios where sources and target samples are collected with space-time correlation. Real data experiments also confirmed the effectiveness of SourceID-NMF in tracking microbial sources.

## 2 Materials and methods

Following FEAST, we assume that a microbial (target) sample is a mixture of several (known and unknown) microbial sources, our goal is to estimate the proportion of each source in the sample. Let $W\in R^{N\;\times \;K}$ and $X\in R^{N\;\times \;1}$ be two matrices recording the taxa abundance in *K* sources and the target sample, respectively, where *N* is the total number of taxa. Thus, *W_ij_* and *X_i_* denote the abundance of a taxon *i* in source *j* and the target sample, respectively. We can estimate the relative abundance of each source in the sample by solving a linear programming problem, i.e. *X* = *WH*, where the solution $H\in R^{K\;\times \;1}$ indicates the proportions of sources in the sample. However, in practice, we only observe the taxa abundance in the target sample without knowing the source type, source contribution, and the taxa abundance in each source. Thus, in addition to the observed taxa abundance in the target sample (i.e. *X*), available microbiome that are related to the target sample are used as reference sources. Let $Y\in R^{N\;\times \;K}$ represent the relative taxa abundance observed in the reference sources. Because the taxa distribution of each microbial source in the target sample may differ from the given reference, using the observed data *Y* to replace *W* directly may incur bias. Thus, given the observed taxa abundance in the target sample *X* and reference sources *Y*, our goal is to estimate *H* and *W* simultaneously. Let $||\cdot |{|}_{F}^{2}$ be the Frobenius Norm. We formulate the following optimization problem to estimate *W* and *H*.

(1) $$\begin{array}{c} \underset{W,H}{\arg\;\min}\frac{1}{2}{\left\|X\;-\;WH\right\|}_{F}^{2}\;+\;\frac{1}{2}{\left\|W\;-\;Y\right\|}_{F}^{2}\\ s.t.\;W\;\geq \;0,H\;\geq \;0,\sum_{j}^{K}H_{j}=1,\sum_{i}^{N}W_{ij}=1{|}_{j=1:K} \end{array}$$

**Figure 1.**
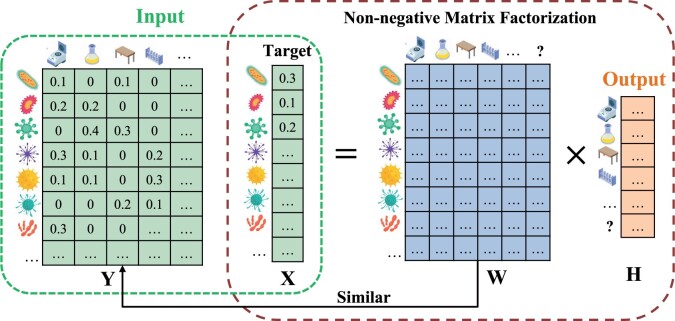
Illustration of the non-negative matrix factorization model for microbial source tracking. The model takes two input matrices in green, representing the relative abundance of taxa in the possible sources and the target sample, respectively. It outputs the proportion of each source present in the target sample. The target sample matrix is factorized into two matrices: the blue matrix, having similar taxa abundance to the reference source matrix, and the orange matrix, indicating the proportion of each source in the target sample.

The problem can be viewed as a non-negative matrix factorization (NMF) problem (i.e. *X* = *WH*), with an added constraint that the taxa compositions in actual sources (*W*) should be similar to the observed taxa compositions in reference sources (*Y*). Figure 1 sketches the problem formulation and the meaning of each component. Because unobserved sources may exist in the sample, we extend *Y*, *W*, and *H* to be $Y\in R^{N\;\times \;(K\;+\;1)},\;W\in R^{N\;\times \;(K\;+\;1)}$, and $H\in R^{(K\;+\;1)\;\times \;1}$ for estimating the total abundance of the unknown sources as well. Since the taxa abundance in the unknown source is missing (i.e. the last column of *Y*), we only force *W* being similar to *Y* in the first *K* columns by introducing a weight matrix $A\in R^{N\;\times \;(K\;+\;1)}$ in the second item $\frac{1}{2}{\left\|W\;-\;Y\right\|}_{F}^{2}$. The values of the first *K* columns in *A* are 1 while the last column contains all 0. Let ○ denotes the Hadamard product operator, problem (1) is rewritten as:

(2) $$\begin{array}{c} \underset{W,H}{\arg\;\min}\frac{1}{2}{\left\|X\;-\;WH\right\|}_{F}^{2}\;+\;\frac{1}{2}{\left\|A○(W\;-\;Y)\right\|}_{F}^{2}\\ s.t.\;W\;\geq \;0,H\;\geq \;0,\sum_{j}^{K\;+\;1}H_{j}=1,\sum_{i}^{N}W_{ij}=1{|}_{j=1:K\;+\;1} \end{array}$$

Since simultaneous optimization of *W* and *H* in problem (2) is nontrivial, we employ the alternating direction method of multipliers (ADMM) algorithm Boyd (2010) to iteratively update the variables until convergence. In the subsequent section, we will present a concise overview of the optimization process. A more detailed description is presented in Supplementary Section S1. Based on the ADMM algorithm, problem (2) can be rewritten as problem (3), which introduces new variables and constraints.

(3) $$\begin{array}{c} \underset{W,H,W^{+},H^{+}}{\arg\;\min}\frac{1}{2}{\left\|X\;-\;WH\right\|}_{F}^{2}\;+\;\frac{1}{2}{\left\|A○(W^{+}\;-\;Y)\right\|}_{F}^{2}\\ s.t.\;W=W^{+},H=H^{+},W^{+}\;\geq \;0,H^{+}\;\geq \;0,\\ \sum_{j}^{K\;+\;1}H_{j}^{+}=1,\sum_{i}^{N}W_{ij}^{+}=1{|}_{j=1:K\;+\;1} \end{array}$$

In problem (3), *H*^+^ and *W*^+^ are the non-negative and normalized versions of *H* and *W*. Thus, we use *H*^+^ as the estimated source proportions. To solve this problem, the augmented Lagrangian function of the problem, without considering the non-negative and normalized constraints on *H*^+^ and *W*^+^, is provided as below:

(4) $$\begin{array}{cc} & L(W,H,W^{+},H^{+},\alpha_{W},\alpha_{H})=\frac{1}{2}{\left\|X\;-\;WH\right\|}_{F}^{2}\;+\;\\ & \frac{1}{2}{\left\|A○(W^{+}\;-\;Y)\right\|}_{F}^{2}\;+\;\;<\;\alpha_{W},W\;-\;W^{+}\;>\;\;+\;\\ & \frac{\rho }{2}{\left\|W\;-\;W^{+}\right\|}_{F}^{2}\;+\;\;<\;\alpha_{H},H\;-\;H^{+}\;>\;\;+\;\frac{\rho }{2}{\left\|H\;-\;H^{+}\right\|}_{F}^{2} \end{array}$$

where *α_W_* and *α_H_* are the dual variables, *ρ* (1 by default) is the penalty parameter, and  < ·,· >  is the inner product operator. ADMM updates four primal variables and two dual variables separately at each iteration until convergence. In the (t + 1)th iteration, the variables are updated by minimizing the augmented Lagrangian function (4) while fixing the values of the other variables. The details of the variable updates in order are shown as below:

 H(t + 1)←(W(t)TW(t) + ρI)−1(W(t)TX + ρH+(t) − αH (t)) W(t + 1)←(ρW+(t) − αW(t) + XH(t + 1)T)(H(t + 1)H(t + 1)T + ρI)−1 D←Djj=∑iWij(t + 1) W(t + 1)←W(t + 1)D−1 H(t + 1)←DH(t + 1) H+(t + 1)←arg min∑jK + 1Hj+=1H+ ≥ 0,L(W(t + 1),W+(t),H(t + 1),H+,αW (t),αH (t)) W+(t + 1)←arg min∑iNWij+=1W+ ≥ 0,L( W(t + 1),W+,H(t + 1),H+(t + 1),αW (t),αH (t)) αW(t + 1)←αW(t) + ρ(W(t + 1) − W+(t + 1)) αH(t + 1)←αH(t) + ρ(H(t + 1) − H+(t + 1))

In this optimization, the updates of $H^{{+}^{\left(t\;+\;1\right)}}$ and $W^{{+}^{\left(t\;+\;1\right)}}$ cannot be written by the explicit method due to the normalized constraints. Thus, we applied the Lagrange multiplier theorem and the Water-filling algorithm (Boyd and Vandenberghe 2004) to update *W*^+^ and *H*^+^ in each iteration. Using the Lagrange multiplier theorem and the KKT condition, we can derive the value of $W^{{+}^{\left(t\;+\;1\right)}}$ and $H^{{+}^{\left(t\;+\;1\right)}}$ by $W_{ij}^{{+}^{\left(t\;+\;1\right)}}=\max(0,\frac{A_{ij}^{2}Y_{ij}\;+\;\alpha_{W}{\;}_{ij}^{\left(t\right)}\;+\;\rho W_{ij}^{\left(t\;+\;1\right)}\;-\;\beta_{j}}{A_{ij}^{2}\;+\;\rho })$ and $H_{i}^{{+}^{\left(t\;+\;1\right)}}=\max(0,\;\frac{\alpha_{H}{\;}_{i}^{\left(t\right)}\;+\;\rho H_{i}^{\left(t\;+\;1\right)}\;-\;\beta }{\rho })$. The *β_j_* and *β* are the Lagrange multipliers and can be inferred easily (Supplementary Section 1).

Using the update rules, we can estimate the proportion of each source by iteratively updating the variables until the convergence condition [as shown in Formula (5)] is satisfied.

(5) $$\frac{\left|L^{\left(t\right)}\;-\;L^{\left(t\;+\;1\right)}\right|}{\left|L^{\left(t\right)}\right|}\;\leq \;\mathrm{threshold}$$

where $L^{\left(t\right)}$ is the value of the Equation (4) given the variables at the *t*th iteration. To avoid infinite iteration, we also set a maximum number of iterations. In this paper, we use $10^{\;-\;6}$ as the threshold and 2000 as the maximum iteration number.

## 3 Results

**Figure 2.**
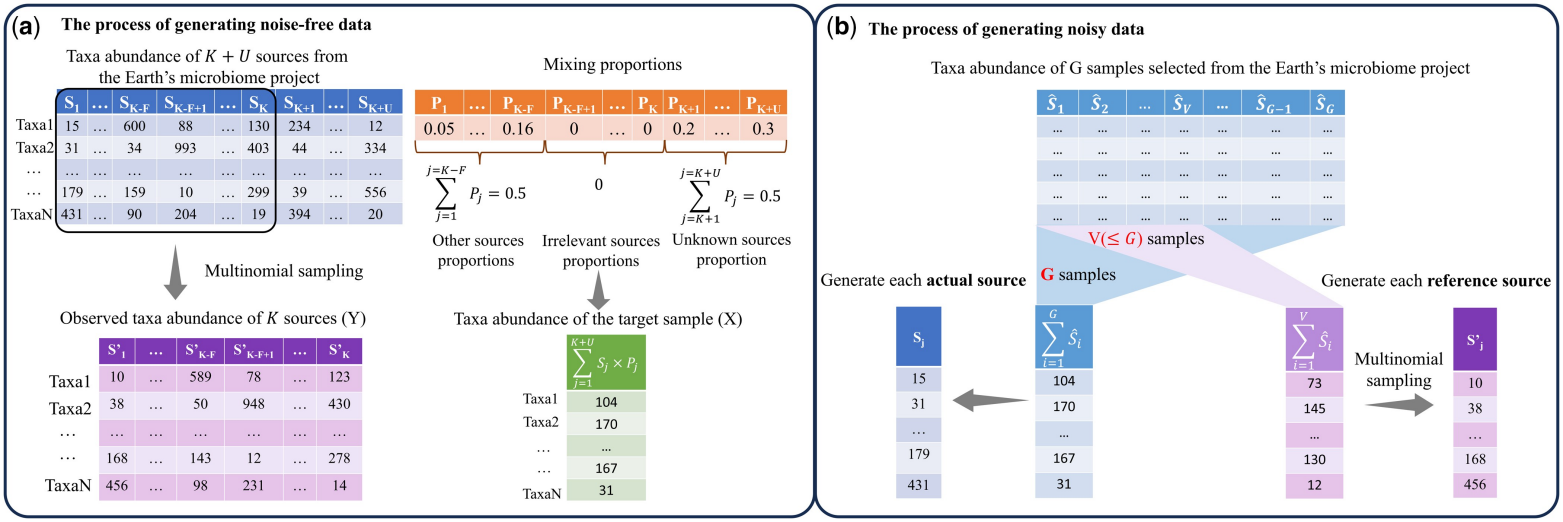
Example of generating two types of simulated data. (a) Generation of the noise-free simulated data with varying inter-source divergences. The blue and orange tables represent taxa abundance of *K *+* U* sources with a varying inter-source divergence from the Earth’s Microbiome Project and their mixing proportions. *F* sources are designated as irrelevant sources (labeled $S_{K-F+1}$ to *S_K_*), while *U* sources (*S_K_* to $S_{K+U}$) are unknown sources with a total proportion of 0.5. The purple table at the bottom shows the observed taxa abundance (*Y*) of *K* sources generated through multinomial distributions from the top blue table. The green table represents the taxa abundance of the generated target sample, obtained by mixing the taxa abundance of the *K *+* U* sources according to the proportions specified in the orange table. (b) The process generating noisy data for amplifying the disparity between observed reference sources and true sources mixed in the target sample. There are two main operations. First, the taxa abundance of each actual source was created by combining *G* samples (light blue table) from the Earth’s Microbiome Project. Second, each observed source was derived by taking the combined taxa abundance of *V* out of *G* samples (V≤G). Decreasing the value of *V* allows for the generation of simulated data with high levels of disparity between observed and mixing sources.

To evaluate the performance of SourceID-NMF, we conducted a series of experiments using both simulated and real data. First, we generated two types of simulated data to mimic challenging cases in real-life samples, including (i) sources with highly similar taxa distributions, (ii) incomplete sources, (iii) irrelevant sources, (iv) low abundance sources, and (v) noisy data in sources. These data allow us to quantify the source tracking performance under different scenarios. The first type of simulated data covers cases (i) to (iv). The second type of simulated data has increased difficulty by introducing noises in each source [challenging case (v)]. Specifically, we amplify the disparity between the observed reference sources data (*Y*) and the actual mixing sources data (*W*). In these two types of simulated data, we intentionally included multiple unknown and irrelevant sources to create a realistic scenario for source tracking. Figure 2 illustrates the process of generating the simulated data. And we also conducted running time and memory usage analysis using the simulated data in the Supplementary Section S2. Then, in the real data experiments, we applied SourceID-NMF to real data sampled from indoor environments (e.g. office buildings, hospitals, and research labs) with surface contamination (Knights *et al.* 2011) and from infants of the Neonatal Intensive Care Unit (NICU) (Brooks *et al.* 2014). In the indoor environmental dataset, the reference source data was obtained from a public database, whereas the target samples were independently sequenced. Consequently, there was no temporal or spatial correlation between them, which limited the accuracy of source tracking to a rough estimate. In the infant-related dataset, the reference source data and the target samples were collected in the same NICU at similar times.

We compared SourceID-NMF with two widely used tools for microbial source tracking: FEAST and SourceTracker. These tools employ probabilistic frameworks to estimate the proportions of sources in a microbial sample. All tools take the taxa abundance in reference sources and the target sample as input and provide the estimated proportion of these sources [H in Equation (2)]. Thus, our evaluation focuses on H, the estimated proportion of each reference source in the target sample. We employed the Jensen-Shannon divergence (JSD) and the Pearson correlation coefficient (PCC) as the primary evaluation metrics in this paper. The JSD, which ranges from 0 to 1, quantifies the relative differences between the estimation and the ground truth directly. It can be calculated using the formula $\mathrm{JSD}(E||T)=\frac{1}{2}KL(E||M)\;+\;\frac{1}{2}KL(T||M)$, where *E* and *T* represent two distributions, and *M* is the mean of *E* and *T*. The Kullback–Leibler divergence (*KL*) is used in this calculation. The PCC, ranging from −1 to 1, focuses on capturing the trend of the source proportions. A small difference between the estimation and the ground truth leads to a low JSD value, whereas a similar trend in the estimation corresponds to a high PCC value. Our empirical testing indicated that a JSD value exceeding 0.3 and a PCC value below 0.6 can be considered significant indicators of substantial differences in proportion values and trends, respectively. It is important to note that the PCC primarily focuses on capturing the trend of source proportions, which may result in high PCC values even for highly different proportion distributions with similar trends. For example, the proportion distributions {0.1, 0.2, 0.3, 0.4} and {0.01, 0.05, 0.1, 0.84} yield a PCC value of 0.83 despite their notable difference, with a JSD value of about 0.33.

### 3.1 Experiments on simulated data

#### 3.1.1 Generating simulated data

We generated the simulated data following the approach in FEAST, which essentially simulates the process of generating a target sample. And we used the microbial data from the Earth’s microbiome project (Thompson *et al.* 2017) to simulate the data following FEAST. A table recording the taxa abundance in each microbial source in the project can be found in our GitHub repository. Based on the table, there are a total of 2000 microbial sources encompassing 53 355 taxa (without filtering any low abundance taxa). On average, each source detects around 702 taxa, with a minimum of 14 and a maximum of 2763. The standard deviation for the taxa count is approximately 645. Furthermore, we assessed the alpha diversity using two metrics: “Shannon Entropy” and “Observed OTUs.” The distribution of the alpha diversity can be found in Supplementary Fig. S12. Among all the sources with known taxa abundance in the table, we selected *K *+* U* sources, designating *U* sources as unknown, to generate a target sample. Specifically, the *K *+* U* sources comprise *W* in Equation (2), whose taxa abundance values are not observable to the source tracking tools. To create a realistic yet challenging scenario for source tracking, we randomly selected a specific set of *F* sources from the *K* sources and assigned zero proportions to them. This means that these *F* sources do not contribute to the target sample and are therefore referred to as irrelevant sources. We then applied the Pareto distribution to generate the random proportion (denoted by *p_j_*) of other sources in the target sample. The orange table in Fig. 2a illustrates the mixing proportions of the *K *+* U* sources in the target sample. Let $S_{j}\in R^{N\;\times \;1}$ be the abundance vector for *N* taxa in source *j* as shown in the blue table. We created the taxa abundance for the target sample by summing the product of *S_j_* and *p_j_* for the *K *+* U* sources (green table). For each actual source *S_j_* (exclude the *U* unknown sources), we employed a multinomial distribution to generate an observed taxa abundance vector for a reference source $S_{j}^{\prime }\in R^{N\;\times \;1}$ (the purple table in Fig. 2a). Finally, the taxa abundance of the target sample and the simulated taxa abundance (S′) of the *K* sources were utilized as input (*X* and *Y*) for all tools in the experiments.

We employed the above simulated process to generate the data by incorporating different inter-source divergences, measured by JSD. To generate target samples using sources of varying average inter-source JSD, we employed a heuristic process. First, we calculated the inter-source divergence between the 2000 sources in the taxa abundance profiling table. Then, for a desired JSD value (e.g. 0.6 JSD), we clustered all pairs of sources with JSD falling within a predefined range (e.g. 0.55 JSD to 0.65 JSD for 0.6). Of each cluster, the most frequent source (i.e. the source leading to the highest number of pairs with the desired JSD) was selected as the first source. Then, of the remaining sources in this cluster, we picked one with two conditions: (i) its inter-source JSD with all the previously picked ones fell into the given range; (ii) it has high frequency in this cluster (same consideration as the first source). This process was repeated until we identified a set of 20 sources. In cases where no remaining source met the requirement for inter-source divergence to the selected sources, we relaxed the range. In total, we selected 12 sets of sources, each comprising 20 sources, with average JSD values ranging from approximately 0.3 to 0.8. Supplementary Fig. S13 shows the pair-wise inter-source divergence for each set. Each set of 20 sources were randomly divided into 5 unknown sources and 15 reference (observed) sources, with 5 of the reference sources being irrelevant (with zero proportions). Then, we created 9 simulated target samples for each set of sources, with the total proportion of the 5 unknown sources varying from 0.1 to 0.9, and assigned random proportions to the remaining 10 sources. For each set of sources, we repeated this generation process three times. Thus, we generated 12 × 9 × 3=324 target samples in total. Since all the tools can only provide an estimation of the total unknown proportion in their output, our evaluation solely focuses on the total unknown proportion instead of individual proportions for each unknown source.

In real-life data, the observed taxa abundance of available sources may not be highly similar to their corresponding contributions in the target sample. For example, due to the difficulty of obtaining closely related source data, some of the selected sources are from public databases with very different sampling and sequencing conditions. To mimic this scenario, we generated the simulated data with more noises by amplifying the disparity between the actual and observed sources (*S* and S′). There are two main differences compared to generating the noise-free simulated data, as shown in Fig. 2b. First, the taxa abundance of each actual source was created by combining the taxa abundance of *G* randomly selected samples from the Earth’s microbiome project ($S_{j}=\sum_{i=1}^{G}{\hat{S}}_{i}$), where ${\hat{S}}_{i}$ represents the taxa abundance of one of the *G* samples. Then, the taxa abundance of the target sample (X) was generated using these created sources. Second, the taxa abundance of each observed source was derived from a different multinomial distribution. This distribution was created by combining the taxa abundance of V( ≤ G) samples ($\sum_{i=1}^{V}{\hat{S}}_{i}$) selected from the *G* samples. By varying the value of *V*, we generated a series of observed taxa abundances for the reference sources (Y). The smaller the number of samples (*V*) used to create the reference sources, the greater the disparity between the actual and observed sources. The selected samples from the Earth’s microbiome project exhibited an average pairwise JSD value of approximately 0.8 following FEAST. Highly dissimilar samples were deliberately chosen to create large disparity between W and Y as the value of *V* varied. Following the approach in FEAST, we used *G *=* *10 to create 10 different sources. The 10 created sources are used as known sources. And we also selected 10 samples from the project directly as 5 irrelevant sources and 5 unknown sources. Using the 20 sources (inter-source divergence of about 0.8 JSD), we created 9 target samples with the total proportion of unknown sources ranging from 0.1 to 0.9. We varied *V* from 1 to 10 to generate 10 observed data (Y) with different levels of noises. Each target sample and each observed data (9 × 10 experiments in total) are then used as inputs for all tools.

#### 3.1.2 Impact of inter-source similarity on source tracking

**Figure 3.**
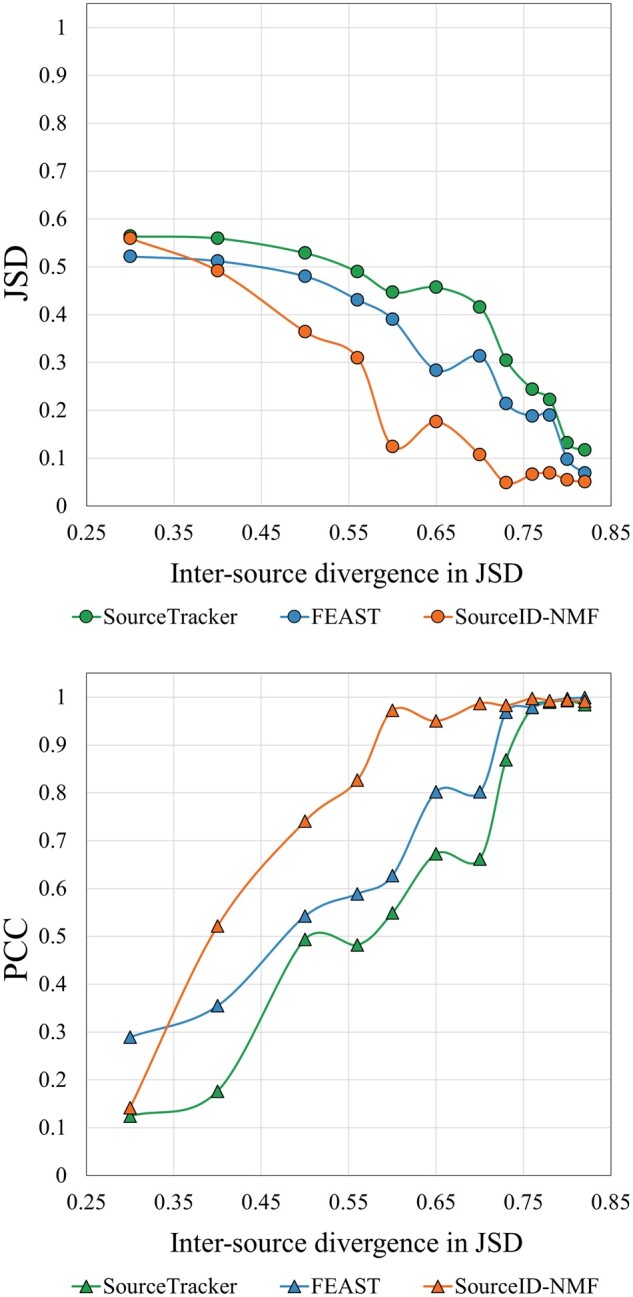
Performance of three tools on simulated datasets with varying inter-source divergence. *X*-axis: averaged pairwise divergence between 20 sources, measured by JSD on taxa abundance. Left to right: increased inter-source divergence. *Y*-axis in two plots: JSD and PCC value between the estimated and the true source proportions (all 20 sources including unknown). Each point represents the average result from 9 × 3 = 27 simulated target samples with the total proportion of unknown sources ranging from 0.1 to 0.9. Supplementary Fig. S11 shows the precision and recall of the identified sources.

When some sources share high composition similarity, it becomes harder to track their contributions accurately. In this experiment, our goal is to evaluate the performance of SourceID-NMF in this case. We tested three tools on the simulated data (less noises) with different inter-source divergences and summarized their performance in Fig. 3. In Fig. 3, we present the performance comparison of three tools under two evaluation metrics JSD and PCC. The *X*-axis represents the average pairwise inter-source similarity quantified by JSD, while the *Y*-axis indicates the JSD values (or the PCC) of source proportions between the estimation and the ground truth. Each point represents the JSD value (or the PCC) between the estimated source proportions and the ground truth given the average pairwise source divergence (*X*-axis). It is clear that with the increase of the inter-source divergence, the source tracking performance becomes more accurate for all three tools. Among the three tools, FEAST and SourceID-NMF outperform SourceTracker due to their lower JSD values and higher PCC values. SourceID-NMF consistently exhibits the lowest JSD and highest PCC values in estimating source proportions for datasets with an inter-source divergence above 0.50 JSD. Notably, SourceID-NMF demonstrates a highly accurate estimation of source proportions when the inter-source divergence reaches a JSD value of 0.8, yielding negligible disparities between the estimation and the ground truth. However, when the inter-source divergence has JSD values below 0.5, all the tools have comparable performance, as they struggled to accurately estimate proportions for similar sources.

#### 3.1.3 Accuracy of tracking unknown sources, irrelevant sources, and low abundance sources

**Figure 4.**
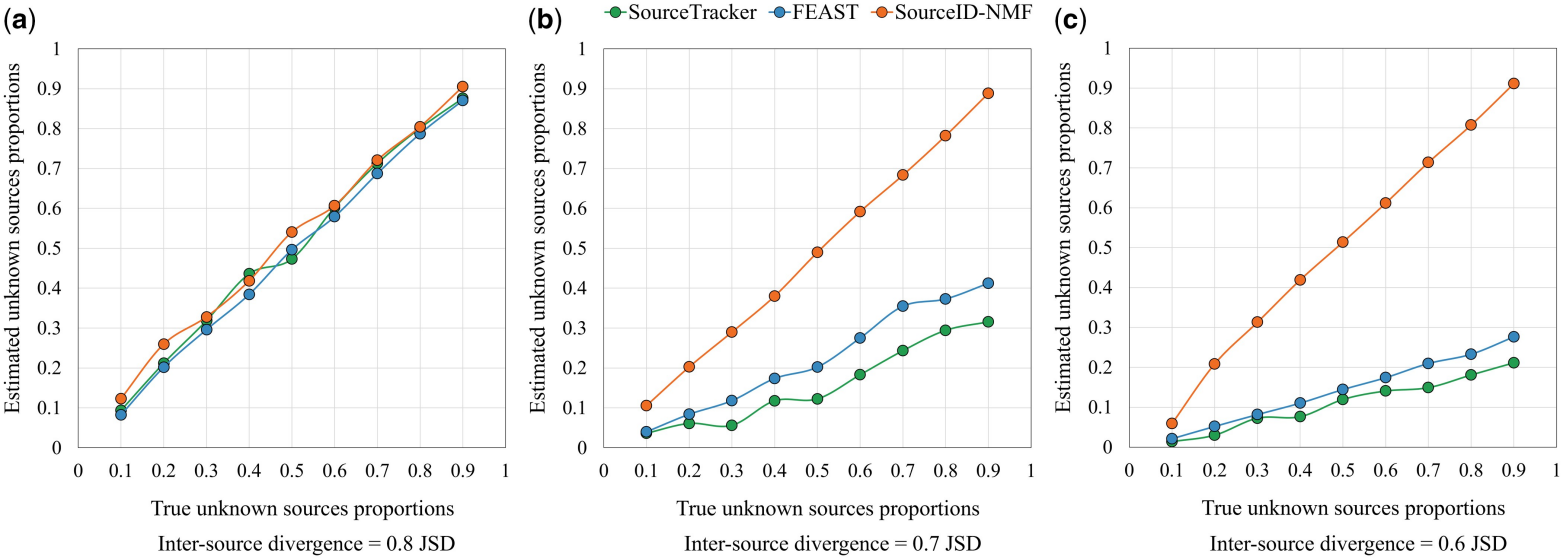
The estimated proportion of the unknown source by three tools on the simulated data with the inter-source divergence in JSD of (a) 0.8, (b) 0.7, and (c) 0.6 in Fig. 3, respectively. The *X*-axis represents the true total proportion of the unknown sources, from 0.1 to 0.9.

The previous analysis demonstrates the overall performance of the three tools on data with different inter-source divergence. In this section, we further investigate the tools’ performance by analyzing the results in Fig. 3 with respect to the estimation of unknown sources, the ability to identify irrelevant sources, and the sensitivity to detect sources with low proportions. In Fig. 4, we plotted the true unknown source proportions (x-axis) and the estimated unknown source proportions using datasets with different inter-source divergences (about 0.6, 0.7, and 0.8 JSD). When the inter-source divergence is high (0.8 JSD), all tools display curves that closely align with the diagonal line, indicating precise estimation of the unknown source proportions. As the inter-source divergence decreases, SourceID-NMF consistently provides accurate estimations. In contrast, SourceTracker and FEAST significantly underestimated the proportions of the unknown sources, as evidenced by their curves approaching the x-axis. This demonstrates the superiority of SourceID-NMF in accurately estimating the proportion of unknown sources.

**Figure 5.**
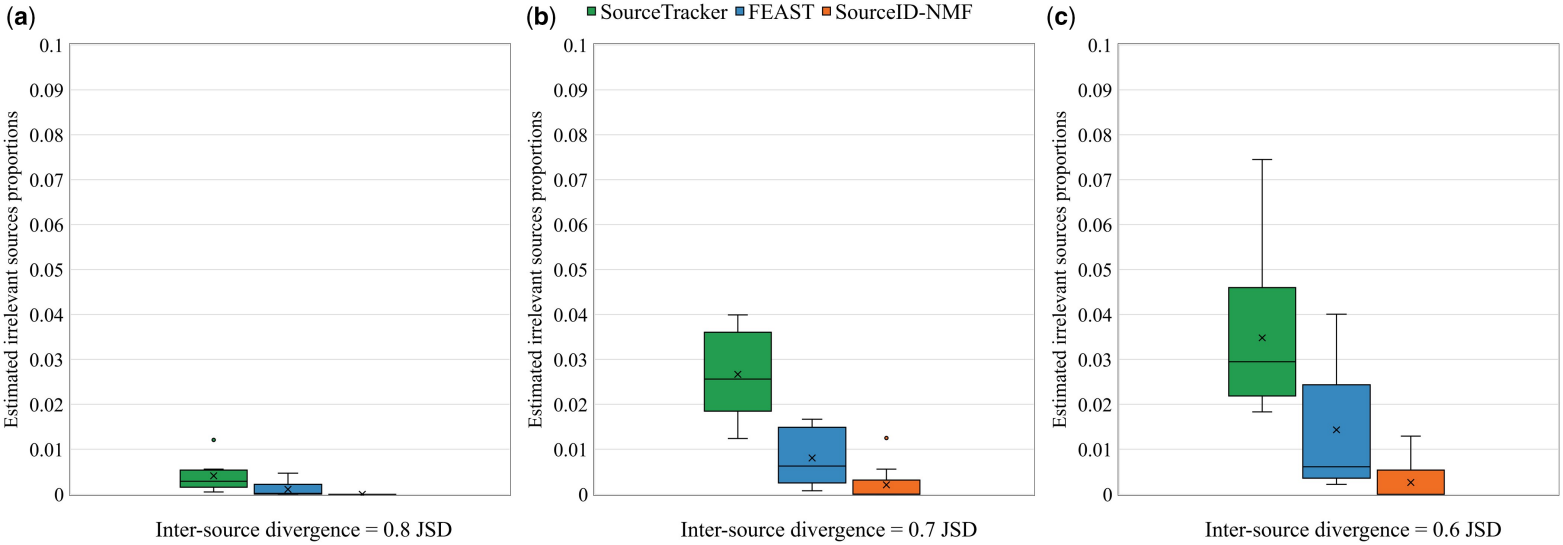
The boxplots of the estimated proportions for the irrelevant sources (zero proportions) by three tools on the simulated data with the inter-source divergence in JSD of (a) 0.8, (b) 0.7, and (c) 0.6 in Fig. 3, respectively.

Then, we examined the estimated proportions of irrelevant sources from the three tools on the same datasets. Figure 5 displays the boxplots of the estimated proportions of irrelevant sources on the datasets with inter-source divergence of 0.6, 0.7, and 0.8 JSD. As shown in Fig. 5, the estimated proportions by SourceID-NMF for irrelevant sources are closer to zero with smaller variability compared to other tools. With the decrease of the inter-source divergence, all three tools exhibit an increase in estimated proportions for irrelevant sources. However, SourceID-NMF still maintains a very low false estimation for those irrelevant sources, demonstrating its robustness against irrelevant sources. In addition, we investigated the impact of the number of irrelevant sources on SourceID-NMF. Details are presented in Supplementary Section S4.

**Figure 6.**
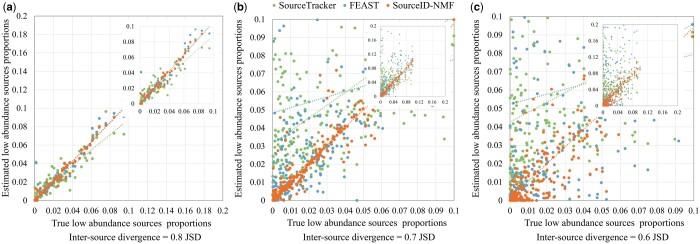
The scatter plots of the estimated proportions for the low abundance sources (below 0.1 proportions) by three tools on the simulated data with the inter-source divergence in JSD of (a) 0.8, (b) 0.7, and (c) 0.6 in Fig. 3, respectively. The *X*-axis and *Y*-axis represent the true and estimated proportions of the low abundance sources, respectively. Each line represents the fitting line of the corresponding scatter points. A boxplot showing the differences is presented in Supplementary Fig. S14.

Another major challenge in source tracking is to trace the source with very low abundance in the target sample. Thus, we investigated the detection of these sources (proportion below 0.1) and plotted the scatter points and fitting lines for their estimated and true proportions, as shown in Fig. 6. When the inter-source divergence is high, the scatter points of the three tools predominantly align along the diagonal. As the inter-source divergence decreases, the scatter points exhibit a more diverse distribution as expected. We found that all three tools exhibit a high sensitivity in detecting low abundance sources, despite the potential for over or underestimation of the proportions. However, SourceID-NMF maintains a higher accuracy in estimating the proportions for low abundance sources, as demonstrated by the diagonal fitting lines of the scatter points in the three plots. Conversely, FEAST and SourceTracker overestimated the proportions of more sources, which is consistent with the previous analysis.

Overall, SourceTracker and FEAST tend to overestimate the proportions of reference sources, including irrelevant sources, while underestimating the proportions of unknown sources. In comparison, SourceID-NMF demonstrates superiority in accurately estimating the proportions of unknown sources. Furthermore, when analyzing irrelevant sources and low abundance sources, SourceID-NMF outperforms other tools in identifying irrelevant sources without compromising the accuracy in tracking the low abundance sources.

#### 3.1.4 Impact of the disparity between observed and true source on source tracking

**Figure 7.**
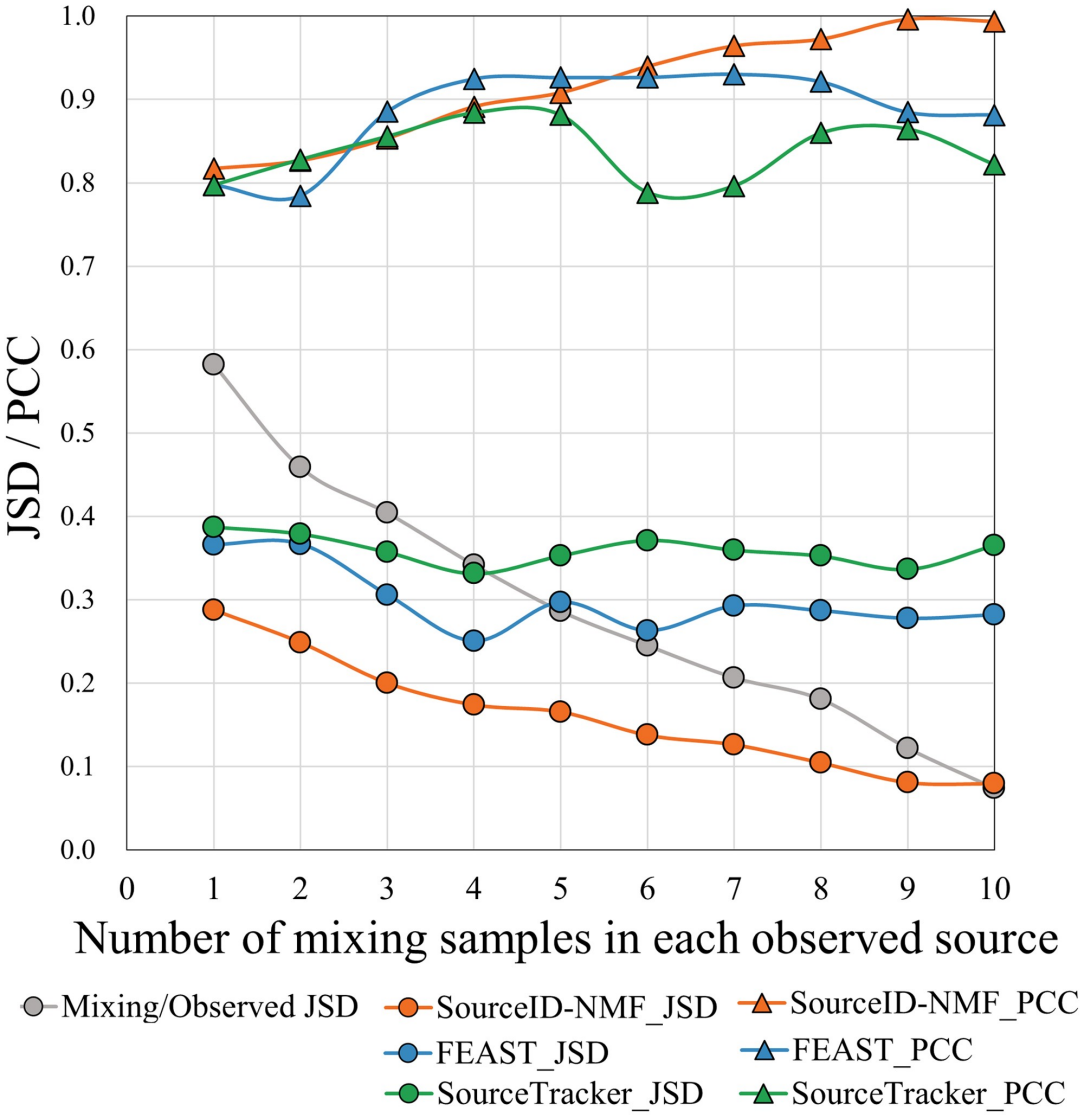
The performance of three tools on simulated datasets with different levels of noises. *X*-axis: the number of mixing samples for generating each observed source (*Y*). The gray points on the curve labeled “Mixing/Observed JSD” denote the JSD value between the actual mixing source (W) and the observed sources (Y). Each circle and triangle point on the curves represents the averaged JSD and PCC values between the estimated and true source proportions across 9 target samples with varying proportions of unknown sources from 0.1 to 0.9.

In this experiment, we tested the robustness of SourceID-NMF when there is a significant disparity in taxa abundance distributions between the true sources mixed in the target sample (*X*) and the observed reference source (*Y*). We applied the three tools to the simulated data with different levels of noises and summarized their results using JSD and PCC in Fig. 7. The gray curve named “Mixing/Observed JSD” represents the average JSD values between the observed reference sources and the true sources mixed in the target sample. The curves with circle and triangle points display the JSD and PCC values between the estimated and actual source proportions. As each true source mixed in X consists of *G* = 10 samples (Fig. 2), the similarity between the observed and true sources increases as we use more samples (i.e. V in Fig. 2) to generate the observed sources, as indicated by the gray curve. SourceID-NMF demonstrates the best performance, with the lowest JSD values and PCC values that are on par with those of competing tools. As expected, when the similarity between the observed and true sources increases, the performance of SourceID-NMF improves in terms of JSD and PCC. However, the performance of SourceTracker and FEAST does not change significantly, which leads to a similar conclusion as observed in FEAST. Importantly, even when there is a significant disparity between the observed and true sources in the target sample (e.g. 0.4 JSD caused by mixing 3 samples in the observed sources), SourceID-NMF maintains good performance and shows a significant improvement over other tools.

### 3.2 Real data experiment

#### 3.2.1 Experiment on indoor environmental samples

**Figure 8.**
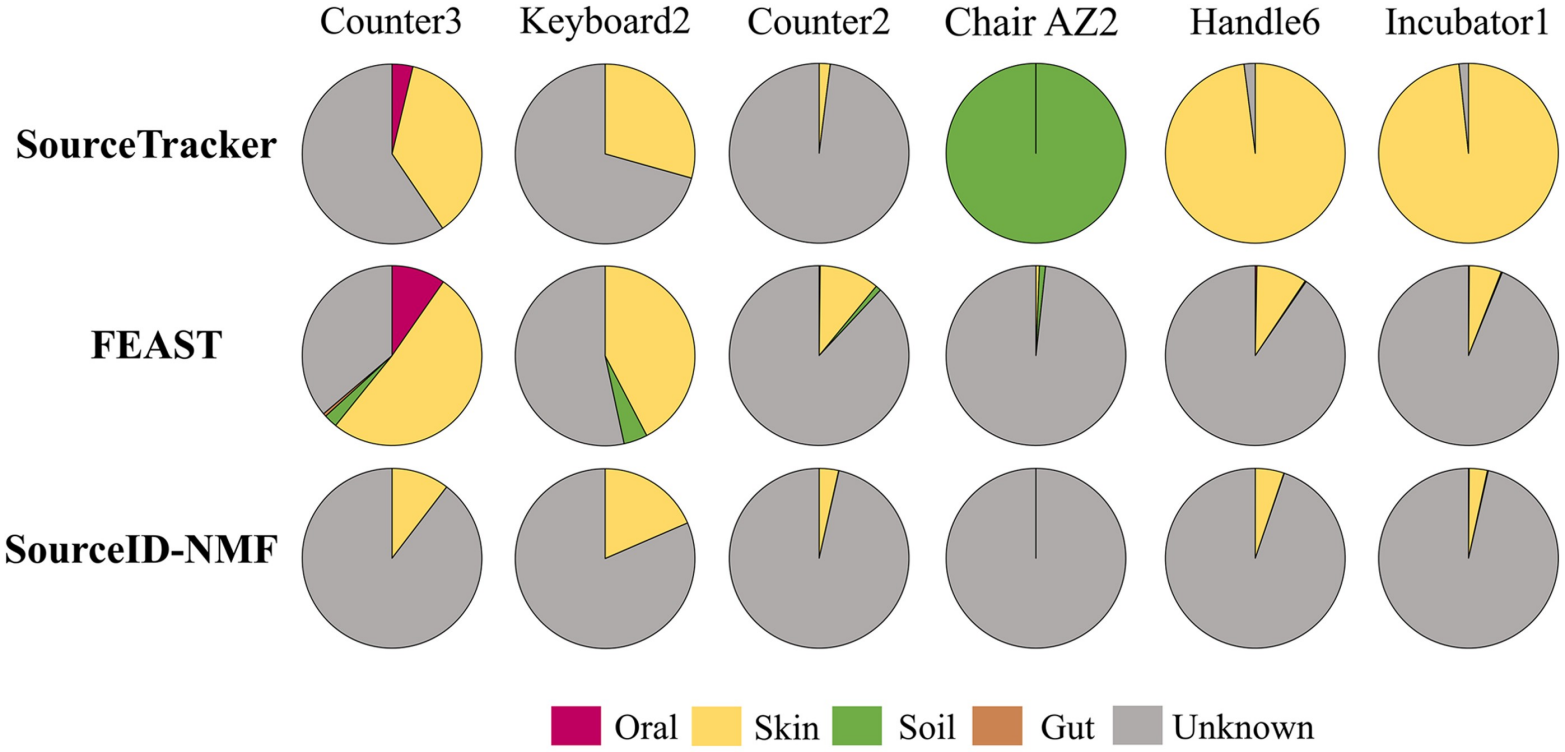
The pie charts of estimated source proportions in six indoor environmental samples using three tools.

In this experiment, we utilized the three tools to track potential sources for indoor environmental samples (Knights *et al.* 2011). There are many samples from the study in Knights *et al.* (2011), and we selected six samples from two counters, a keyboard, a doorknob, an incubator, and an office chair. Among these six samples, three had previously been analyzed using SourceTracker and FEAST. The remaining three samples were chosen due to their higher taxa abundance than other samples. Following (Knights *et al.* 2011, Shenhav *et al.* 2019), we used 180 public microbial samples from gut, oral, skin and soil (45 samples for each), as possible sources for estimating source proportions in the samples. We plotted the pie charts of the estimated source proportions from the tools in Fig. 8.

**Figure 9.**
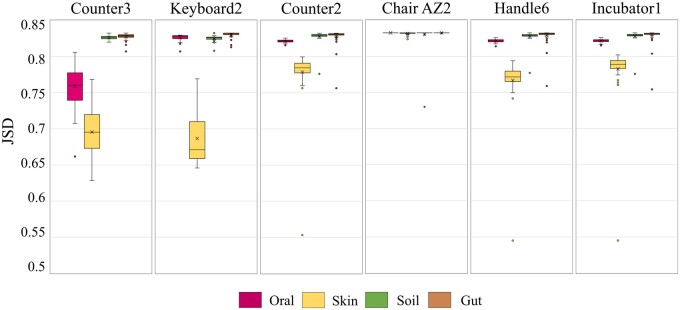
Composition similarity between the 6 target samples and 180 sources, measured using JSD. The 180 sources were categorized into gut, oral, skin, and soil.

When focusing on the identified source types, all three tools identified skin-originated sources in samples collected from counters, keyboards, handles, and incubators. This observation aligns with the expectation that many microbes found on these surfaces originate from the skin. When considering the source proportion, both SourceID-NMF and FEAST produce similar results in the last four samples. However, noticeable differences arise in the proportion estimations of the first two samples, as illustrated in Fig. 8. SourceTracker, on the other hand, exhibits more divergent results from the other two tools in certain samples (e.g. the last three pie charts). It assigned larger proportions to specific sources, while SourceID-NMF and FEAST indicated that most microbes in these samples come from unknown sources. To further investigate the differences in proportion estimation among the three tools, we analyzed the similarity of taxa abundance between these samples and potential sources. We plotted the JSD values of the taxa abundance between the environmental samples and sources using box plots in Fig. 9.

We observed that the JSD values between the samples and all sources consistently exceeded 0.6, with the majority hovering around 0.8. This suggests a significant difference in taxa abundance between the sources and the samples. Notably, in the first sample, the JSD values for skin and oral sources were relatively small compared to other sources. Consequently, SourceTracker and FEAST assigned larger proportions to these two sources. However, considering that the JSD values for these sources remained substantial, SourceID-NMF took a conservative estimation and assigned proportions to the skin sources. In the chair sample, SourceTracker allocated nearly 100% proportion to the soil sources, whereas both FEAST and SourceID-NMF assigned extremely small proportions to the soil sources. While it is reasonable to assume that some microbes in the chair sample might originate from the soil, the high JSD values between the sample and the soil sources indicate that the sample should not be entirely attributed to soil sources. Therefore, the proportion estimation of this sample by FEAST and SourceID-NMF appears to be more reasonable. In conclusion, SourceTracker and FEAST exhibited a tendency to greedily identify more potential sources, whereas SourceID-NMF prioritized estimating sources with higher confidence. Due to the absence of spatial and temporal correlation in the collection of samples and sources, SourceID-NMF assigned larger proportions to the unknown sources compared to the other two tools.

#### 3.2.2 Experiment on tracking potential sources in infants’ fecal samples

In this experiment, we assessed the performance of SourceID-NMF in tracking potential sources of colonizing microbes in the gut environment of infants from the NICU (Brooks *et al.* 2014). The target samples are the fresh fecal samples, while the potential sources were collected from six designated areas within the NICU rooms. Altogether, we have 7 fecal samples and 29 sources from hands, environmental surfaces, incubators, sinks, tubes, and electronics within the NICU.

**Figure 10.**
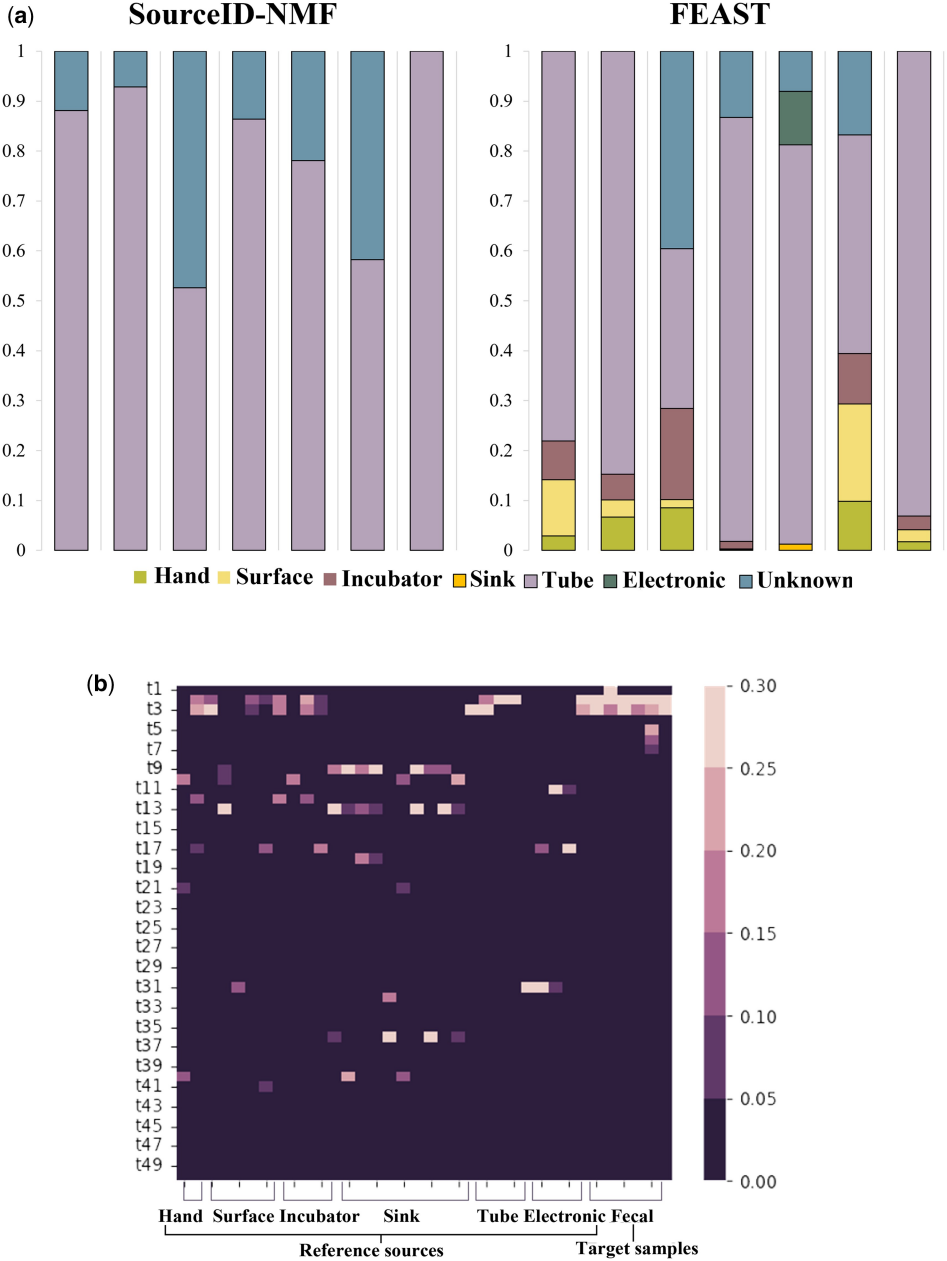
The results of SourceID-NMF and FEAST in tracking potential sources of gut-colonizing microbes in infants from NICUs. (a) The estimated source proportions in the fecal sample. (b) The heat map represents the relative taxa abundance in sources and fecal samples. The rows correspond to the 50 most abundant taxa across different samples, while the columns represent the sources and fecal samples.

Given that FEAST and SourceID-NMF outperform SourceTracker in both the simulated experiments and the real indoor data analysis, we only applied FEAST and SourceID-NMF to this dataset and presented their results in Fig. 10a. Both tools found that the most dominant sources in the samples are from tubes. Because the tubes are situated in the closest proximity to the infants, it is reasonable to expect that the microbes in the samples are mostly from tubes. Besides the tubes, FEAST also identified other sources with small proportions in the fecal samples, while SourceID-NMF did not report other sources. As the previous experiments showed that FEAST may overestimate some sources, we further investigate the difference between the two tools by checking the taxa distributions in sources and infants’ samples. The heatmap in Fig. 10b shows the relative abundance of taxa with high abundances in sources and target samples.

In Fig. 10b, the fecal samples contain enriched taxa represented by the first 6 rows. We observed that the sources from hand, surface, incubator, and tube contain some of the taxa present in the fecal samples, which explains why FEAST identified these sources in the fecal samples. However, sources from hand, surface, and incubator also exhibit other taxa with high abundance (e.g. >10%) that are not detected in the fecal samples. The absence of abundant taxa in the fecal sample explains why SourceID-NMF chose not to consider those sources as existing sources, thereby minimizing the loss during optimization. To further investigate the reason behind the different outputs by FEAST and SourceID-NMF on such data, we conducted two systematic experiments (Supplementary Section 5).

The experiments simulated two scenarios: one where relevant sources had unique taxa absent in the target samples, and another where irrelevant sources shared some taxa with the target samples but also had unique taxa. In the first experiment, we dropped taxa directly from the target sample, allowing more sources to have unique taxa with respect to the target sample. The results showed that when only the top 20% dominant taxa were retained in the target samples, SourceID-NMF regarded some sources irrelevant, while FEAST still output them. This finding aligned with the results from real data experiments. To allow more aggressive prediction of sources, we tested the strategy that removes unique taxa from the reference sources for SourceID-NMF. In the second experiment, we observed that FEAST tended to include more sources if they shared some taxa with the target samples, resulting in the inclusion of more irrelevant sources and underestimation of the proportion of unknown sources. This observation aligned with the results from the simulated experiments (Section 3.1.3). In comparison, SourceID-NMF performed well in estimating the proportions of unknown sources and identifying fewer irrelevant sources with non-zero proportions. For a detailed discussion of these simulated experiments, please refer to the Supplementary Section S5.

## 4 Conclusions

The microbiome of a sampled habitat includes microbial communities from diverse sources, including potential contaminants. Microbial source tracking (MST) is used to identify the contribution of each source to the observed microbiome data, enabling applications such as monitoring microbial contamination in clinical labs and tracing pollution sources in environmental samples. In light of this, we have developed a tool called SourceID-NMF for tracking microbial sources in a given microbial sample. Unlike existing tools that assume a specific distribution of the taxa in a sample, SourceID-NMF introduces a non-negative factorization model to factorize the taxa abundance of a microbial sample into two matrices: one representing the taxa abundance in sources and the other representing the source proportions in the sample.

We compared SourceID-NMF with state-of-the-art tools using both simulated and real data. The simulated experiments with different inter-source divergences clearly demonstrate the advantages of SourceID-NMF in accurately estimating source proportions. The investigation of unknown sources, irrelevant sources, and low abundance sources further demonstrates the superiority of SourceID-NMF. Furthermore, we evaluated the performance of SourceID-NMF when the observed taxa abundance in sources is highly different from the real mixing sources. The results demonstrated the robustness of SourceID-NMF in tracking sources from noise observed data. In addition, we observed that SourceID-NMF demonstrated a tendency to identify potential sources with high confidence or correlation within a sample. This characteristic makes it particularly suitable for scenarios where the target sample and reference sources are collected with space-time correlation. For example, in applications that need accurate identification of true pathogens in clinical samples such as bronchoalveolar lavage (BAL), removing contamination from oral microbiome, tubes, equipment, etc. become very important. When those control samples are provided as reference sources, SourceID-NMF’s high accuracy in source tracking makes it a preferred choice in these scenarios. However, when the target samples and reference sources are unrelated in terms of space-time correlation, the taxa composition between them differs significantly. In such cases, SourceID-NMF provides a rough estimation of the source contributions by assigning larger proportions to the unknown sources.

In this paper, we assume that the taxa proportions observed in a source are similar to the taxa proportions contributed by that source to a target sample. This assumption is also present in models like SourceTracker and FEAST. When the taxa proportions of a contributing source remain relatively stable, our model can efficiently estimate the source proportions. However, if the taxa distribution undergoes significant changes, the observed taxa abundance may differ greatly from the taxa abundance mixed into the target sample from the same sources. This discrepancy can lead to inaccurate estimation of source proportions by our model, as well as other similar models. A recent study (Wang *et al.* 2023) has investigated the impact of ecological dynamics on microbial source tracking models, demonstrating that significant changes in a source pose significant challenges to source tracking. In future work, we will take into account the dynamic changes in the source tracking problem and investigate approaches to enhance the accuracy of source proportion estimation in such scenarios.

## Supplementary Material

btae227_Supplementary_Data

## Data Availability

Data available on request.
