# Supplementary material for: Towards more accurate microbial source tracking via non-negative matrix factorization (NMF)
Source: Bioinformatics. 2024 Jun 28;40(Suppl 1):i68–78. doi: 10.1093/bioinformatics/btae227 (PMC11256951; doi:10.1093/bioinformatics/btae227)
Supplement: btae227_Supplementary_Data [file btae227_supplementary_data.pdf]

Towards more accurate microbial source tracking via non-negative matrix factorization (NMF)

Ziyi Huang, Dehan Cai, and Yanni Sun

Contents

1 Optimizing the NMF problem using ADMM **2**

2 Running time and memory usage analysis **5**

3 Similarity between W and true sources analysis **6**

4 Impact of varying the number of irrelevant sources on performance **6**

5 Further analysis on tracking potential sources in infants' fecal samples **7**

6 Other Supplementary Figures **11**

1 Optimizing the NMF problem using ADMM

We aim to estimate the taxa abundance distributions $W \in \mathbb{R}^{N\times(K+1)}$ and their contributions $H \in \mathbb{R}^{(K+1)\times 1}$ to the target sample by optimizing the following problem. The observed taxa abundance in the target sample is denoted as $X \in \mathbb{R}^{N\times 1}$, and the reference sources are represented by $Y \in \mathbb{R}^{N\times(K+1)}$, along with an indicator matrix $A \in \mathbb{R}^{N\times(K+1)}$. In this optimization problem, our goal is to find the optimal values for W and H that best describe the contributions of the reference sources to the target sample, based on the observed taxa abundance in X and Y.

$$\begin{aligned} &\arg\min_{W,H} \frac{1}{2}\|X - WH\|_F^2 + \frac{1}{2}\|A \circ (W - Y)\|_F^2 \\ &s.t.\ W \geq 0, H \geq 0, \sum_j^{K+1} H_j = 1, \sum_i^N W_{ij} = 1 (j = 1, ..., K+1) \end{aligned} \tag{1}$$

To tackle the challenge of simultaneously optimizing W and H in problem (1), we utilize the alternating direction method of multipliers (ADMM) algorithm [1]. This algorithm allows us to optimize the variables iteratively until convergence. By applying the ADMM algorithm, we introduce three auxiliary variables: $V \in \mathbb{R}^{N\times 1}$, $H^+ \in \mathbb{R}^{(K+1)\times 1}$, and $W^+ \in \mathbb{R}^{N\times(K+1)}$. These variables are subject to the following constraints: $V = WH$, $W = W^+$, $H = H^+$, $\sum_j^{K+1} H_j^+ = 1$, and $\sum_i^N W_{ij}^+ = 1$ for $j = 1, ..., K+1$. This allows us to formulate an equivalent problem (2) to facilitate the optimization process.

$$\begin{aligned} &\arg\min_{V,W,H,W^+,H^+} \frac{1}{2}\|X - V\|_F^2 + \frac{1}{2}\left\|A \circ (W^+ - Y)\right\|_F^2 \\ &s.t.\ V = WH, W = W^+, H = H^+, W^+ \geq 0, H^+ \geq 0, \sum_j^{K+1} H_j^+ = 1, \sum_i^N W_{ij}^+ = 1 (j = 1, ..., K+1) \end{aligned} \tag{2}$$

To decouple the variables W and H and enable separate updates within the ADMM framework, we introduce the auxiliary variable V with the constraint $V = WH$ in problem (2). However, recent studies [2, 3, 4] have shown that splitting V and WH is unnecessary. In fact, it has been demonstrated in [4] that a simpler equivalent problem, without introducing the auxiliary variable V, exhibits convergence. Therefore, to reduce computational complexity, we consider a simpler equivalent problem (3) that does not require the use of the auxiliary variable V.

$$\begin{aligned} &\arg\min_{W,H,W^+,H^+} \frac{1}{2}\|X - WH\|_F^2 + \frac{1}{2}\left\|A \circ (W^+ - Y)\right\|_F^2 \\ &s.t.\ W = W^+, H = H^+, W^+ \geq 0, H^+ \geq 0, \sum_j^{K+1} H_j^+ = 1, \sum_i^N W_{ij}^+ = 1 (j = 1, ..., K+1) \end{aligned} \tag{3}$$

In problem (3), H^+ and W^+ are the non-negative and normalized versions of H and W, respectively. Therefore, we utilize H^+ as the estimated source proportions. To solve this problem, we consider the augmented Lagrangian function of the problem, disregarding the non-negative and normalized constraints on H^+ and W^+. The augmented Lagrangian function is provided below:

$$\begin{aligned} &L(W, H, W^+, H^+, \alpha_W, \alpha_H) = \frac{1}{2}\|X - WH\|_F^2 + \\ &\frac{1}{2}\left\|A \circ (W^+ - Y)\right\|_F^2 + < \alpha_W, W - W^+ > + \\ &\frac{\rho}{2}\left\|W - W^+\right\|_F^2 + < \alpha_H, H - H^+ > + \frac{\rho}{2}\left\|H - H^+\right\|_F^2 \end{aligned} \tag{4}$$

In the augmented Lagrangian function, α_W and α_H represent the dual variables, ρ (defaulting to 1) is the penalty parameter, and $< \cdot, \cdot >$ denotes the inner product operator. The ADMM algorithm updates four primal variables and two dual variables separately at each iteration until convergence. In the $(t+1)$-th iteration, the primal variables are updated by minimizing the augmented Lagrangian function (4) while keeping the values of the other variables fixed, as shown in the formulas (5).

$$H^{(t+1)} = \arg\min_{H} L(W^{(t)}, {W^+}^{(t)}, H, {H^+}^{(t)}, {\alpha_W}^{(t)}, {\alpha_H}^{(t)}) \tag{5a}$$

$$W^{(t+1)} = \arg\min_{W} L(W, {W^+}^{(t)}, H^{(t+1)}, {H^+}^{(t)}, {\alpha_W}^{(t)}, {\alpha_H}^{(t)}) \tag{5b}$$

$$H^{+(t+1)} = \arg\min_{\substack{H^+ \geq 0,\\ \sum_j^{K+1} H_j^+ = 1}} L(W^{(t+1)}, {W^+}^{(t)}, H^{(t+1)}, H^+, {\alpha_W}^{(t)}, {\alpha_H}^{(t)}) \tag{5c}$$

$$W^{+(t+1)} = \arg\min_{\substack{W^+ \geq 0,\\ \sum_i^{N} W_{ij}^+ = 1}} L(W^{(t+1)}, W^+, H^{(t+1)}, {H^+}^{(t+1)}, {\alpha_W}^{(t)}, {\alpha_H}^{(t)}) \tag{5d}$$

$$\alpha_W^{(t+1)} = \alpha_W^{(t)} + \rho(W^{(t+1)} - {W^+}^{(t+1)}) \tag{5e}$$

$$\alpha_H^{(t+1)} = \alpha_H^{(t)} + \rho(H^{(t+1)} - {H^+}^{(t+1)}) \tag{5f}$$

In problems (5a) and (5b), we determine the solutions for W and H by directly equating their derivatives to zero, as shown in Eqn. (6). Solving the equations in (6) allows us to derive closed-form updates for $W^{(t+1)}$ and $H^{(t+1)}$, as demonstrated in Eqn. (7).

$$\frac{\mathrm{d}L(W^{(t)}, {W^+}^{(t)}, H, {H^+}^{(t)}, {\alpha_W}^{(t)}, {\alpha_H}^{(t)})}{\mathrm{d}H} = 0 \tag{6a}$$

$$\frac{\mathrm{d}L(W, {W^+}^{(t)}, H^{(t)}, {H^+}^{(t)}, {\alpha_W}^{(t)}, {\alpha_H}^{(t)})}{\mathrm{d}W} = 0 \tag{6b}$$

$$H^{(t+1)} = ({W^{(t)}}^T W^{(t)} + \rho I)^{-1}({W^{(t)}}^T X + \rho {H^+}^{(t)} - {\alpha_H}^{(t)}) \tag{7a}$$

$$W^{(t+1)} = (\rho {W^+}^{(t)} - \alpha_W^{(t)} + X {H^{(t+1)}}^T)(H^{(t+1)} {H^{(t+1)}}^T + \rho I)^{-1} \tag{7b}$$

In the standard NMF problem, the factorization is not unique, and the matrices $\hat{W} = WD^{-1}$ and $\hat{H} = DH$ (D is a diagonal matrix with arbitrary values) can be used to replace W and H in the component $||X - WH||_F^2$ without changing the loss. However, if we desire normalized matrices in the factorization, we need to introduce normalization constraints on W and H. Since these constraints are primarily applied to the auxiliary variables W^+ and H^+ within the ADMM framework, we include a post-processing step to normalize $W^{(t+1)}$ by $W^{(t+1)}D^{-1}$ before updating the remaining variables. Here, $D \in \mathbb{R}^{(K+1)\times(K+1)}$ is a diagonal matrix with $D_{jj} = \sum_i W^{(t+1)}ij$. By updating $H^{(t+1)}$ as $DH^{(t+1)}$, we ensure that the optimization for the main component $||X - W^{(t+1)}H^{(t+1)}||_F^2$ is almost not affected. Similarly, the optimization of other components, such as $\frac{\rho}{2}||W - W^+||_F^2$, is not compromised because W^+ and H^+ are normalized. It is important to note that this post-processing step facilitates the optimization process, enhancing speed and stability. However, even without this step, the optimization can still yield optimal solutions after convergence.

In problems (5c) and (5d), we employ the Lagrange multiplier theorem and the Water-filling algorithm [5] to derive the update rules for ${W^+}^{(t+1)}$ and ${H^+}^{(t+1)}$. For convenience, we will remove the iteration index for each variable, and the problem formulations of (5c) and (5d) are as follows:

$$\begin{gathered}\arg\min_{H^+} < \alpha_H, H - H^+ > + \frac{\rho}{2}\left\|H - H^+\right\|_F^2 \\ s.t.\ H^+ \geq 0, \sum_j^{K+1} H_j^+ = 1\end{gathered} \tag{8}$$

$$\begin{gathered}\arg\min_{W^+} \frac{1}{2}\left\|A \circ (W^+ - Y)\right\|_F^2 + < \alpha_W, W - W^+ > + \frac{\rho}{2}\left\|W - W^+\right\|_F^2 \\ s.t.\ W^+ \geq 0, \sum_i^{N} W_{ij}^+ = 1 (j = 1, ..., K+1)\end{gathered} \tag{9}$$

Let λ_j $(j = 1, ..., K+1)$ and β be the Lagrange multipliers, the Lagrange function and the Karush-Kuhn-Tucker(KKT) conditions for problem (8) are shown in formulas (10) and (11):

$$L_{H^+} =< \alpha_H, H - H^+ > + \frac{\rho}{2} \left\| H - H^+ \right\|_F^2 - \sum_{j=1}^{K+1} \lambda_j H_j^+ + \beta(\sum_j^{K+1} H_j^+ - 1) \tag{10}$$

$$\begin{cases} \frac{\mathrm{d}L_{H^+}}{\mathrm{d}H_j^+} = -\alpha_{Hj} - \rho(H_i - H_i^+) - \lambda_j + \beta = 0 \; (j = 1 : K+1) \\ \lambda_j H_j^+ = 0 \; (j = 1 : K+1) \\ \lambda_j \geq 0 \; (j = 1 : K+1) \\ H_j^+ \geq 0 \; (j = 1 : K+1) \\ \sum_j^{K+1} H_j^+ - 1 = 0 \end{cases} \tag{11}$$

By solving the formulas in (11), we obtain the following results:

$$\sum_j^{K+1} \max(0, \; \frac{\alpha_{Hj}^{(t)} + \rho H_j^{(t+1)} - \beta}{\rho}) = 1 \tag{12a}$$

$$H_j^+ = \max(0, \; \frac{\alpha_{Hj} + \rho H_j - \beta}{\rho})(j = 1 : K+1) \tag{12b}$$

The equations in (12) have similar formats to the equations in the water-filling algorithm. In the equation (12a), we have a single unknown variable β. We can estimate the optimal value of β by finding the root of the function with respect to it easily, i.e., $f(\beta) = \sum_i^{K+1} \max(0, \; \frac{\alpha_{Hi}^{(t)} + \rho H_i^{(t+1)} - \beta}{\rho}) - 1$. In this paper, the root-finding method 'bisect' from the Scipy Python package [6] is used to find the optimal value of β. Once we obtain the optimal value of β, we can calculate the solution for H^+ using equation (12b).

Following a similar approach, we show the Lagrange function and the KKT conditions for problem (9) in formulas (13) and (14).

$$L_{W^+} = \frac{1}{2} \left\| A \circ (W^+ - Y) \right\|_F^2 + < \alpha_W, W - W^+ > + \frac{\rho}{2} \left\| W - W^+ \right\|_F^2 - \lambda W^+ + \sum_{j=1}^{K+1} \beta_j (\sum_{i=1}^{N} W_{ij}^+ - 1) \tag{13}$$

$$\begin{cases} \frac{\mathrm{d}L_{W^+}}{\mathrm{d}W_{ij}^+} = A_{ij}(W_{ij}^+ - Y_ij) - \alpha_{Wij} - \rho(W_{ij} - W_{ij}^+) - \lambda_{ij} + \beta_j = 0 \; (i = 1 : N, j = 1 : K+1) \\ \lambda_{ij} W_{ij}^+ = 0 \; (i = 1 : N, j = 1 : K+1) \\ \lambda_{ij} \geq 0 \; (i = 1 : N, j = 1 : K+1) \\ W_{ij}^+ \geq 0 \; (i = 1 : N, j = 1 : K+1) \\ \sum_{i=1}^{N} W_{ij}^+ - 1 = 0 \; (j = 1 : K+1) \end{cases} \tag{14}$$

By solving the formulas in (14), we obtain the following results:

$$\sum_i \max(0, \frac{A_{ij}^2 Y_{ij} + \alpha_{Wij} + \rho W_{ij} - \beta_j}{A_{ij}^2 + \rho}) = 1 \; (for \; j = 1 : K) \tag{15a}$$

$$W_{ij}^+ = \max(0, \frac{A_{ij}^2 Y_{ij} + \alpha_{Wij} + \rho W_{ij} - \beta_j}{A_{ij}^2 + \rho}) \; (for \; i = 1 : N, \; j = 1 : K) \tag{15b}$$

Similarly, we can calculate the optimal value for each β_j $(j = 1 : K)$, and then derive the value for W^+. In summary, the update rules for each variable are as follows:

$$H^{(t+1)} \leftarrow (W^{(t)^T} W^{(t)} + \rho I)^{-1} (W^{(t)^T} X + \rho H^{+(t)} - \alpha_H{}^{(t)}) \tag{16a}$$

$$W^{(t+1)} \leftarrow (\rho W^{+(t)} - \alpha_W^{(t)} + X H^{(t+1)^T})(H^{(t+1)} H^{(t+1)^T} + \rho I)^{-1} \tag{16b}$$

$$D \leftarrow D_{jj} = \sum_i W_{ij}^{(t+1)} \tag{16c}$$

$$W^{(t+1)} \leftarrow W^{(t+1)} D^{-1} \tag{16d}$$

$$H^{(t+1)} \leftarrow D H^{(t+1)} \tag{16e}$$

$$H^{+(t+1)} \leftarrow H_j^{+(t+1)} = \max(0, \frac{\alpha_{H_j}^{(t)} + \rho H_j^{(t+1)} - \beta}{\rho}) \tag{16f}$$

$$W^{+(t+1)} \leftarrow W_{ij}^{+(t+1)} = \max(0, \frac{A_{ij}^2 Y_{ij} + \alpha_{W_{ij}}^{(t)} + \rho W_{ij}^{(t+1)} - \beta_j}{A_{ij}^2 + \rho}) \tag{16g}$$

$$\alpha_W^{(t+1)} \leftarrow \alpha_W^{(t)} + \rho (W^{(t+1)} - W^{+(t+1)}) \tag{16h}$$

$$\alpha_H^{(t+1)} \leftarrow \alpha_H^{(t)} + \rho (H^{(t+1)} - H^{+(t+1)}) \tag{16i}$$

We initialize matrices W and W^+ by directly assigning the values of Y to them. However, since the taxa abundance distribution of the unknown source is missing, we initialize the corresponding column in W and W^+ using the normalized expression $max(X - \sum_j^K Y_i j, 1e-16)$, motivated by FEAST. This approach allows us to allocate the abundance of unique taxa observed in the target sample X to the unknown source, resulting in more accurate estimation of the unknown source proportion. We use $1/(K+1)$ to initialize matrices H and H^+. The dual variables α_W and α_H are initialized as zero. While random initialization can provide estimates of source proportions with a certain level of accuracy, we have discovered that our initialization strategy improves the accuracy of source proportion estimation. Using the update rules, we can estimate the proportion of each source by iteratively updating the variables until the convergence condition (as shown in Formula (17)) is satisfied.

$$\frac{|L^{(t)} - L^{(t+1)}|}{|L^{(t)}|} \leq threshold \tag{17}$$

where $L^{(t)}$ is the value of the equation (4) given the variables at the t-th iteration. To avoid infinite iteration, we also set a maximum number of iterations. In this paper, we use 10^{-6} as the threshold and 2,000 as the maximum iteration number. In Figure S10, we illustrated the evolution of loss on various datasets. These figures demonstrate the convergence efficiency when applying ADMM to solve our NMF problem.

2 Running time and memory usage analysis

In this section, we focused on evaluating the running time and memory usage of SourceID-NMF. In the optimization of SourceID-NMF, the primary computations involve the inversion of a $(K+1) \times (K+1)$ matrix and the matrix multiplication $W^T X$ (or XH^T). These computations have time complexities of $O(K^3)$ and $O(NK)$, respectively. Here, K represents the number of reference sources used as input, and N corresponds to the number of taxa. When the number of input reference sources (K) is large, the computation of the matrix inversion can be particularly time-consuming. Thus, we conducted simulated experiments using various number of reference sources as input and recorded the running time and memory usage. As demonstrated in the FEAST study, FEAST outperforms SourceTracker in terms of estimating source proportions and also exhibits faster runtime. Therefore, we specifically compared SourceID-NMF and FEAST in terms of their running time and memory usage. We conducted simulated experiments on an HPCC CentOS 7.6.1810 node equipped with 2.70GHz Intel(R) Xeon(R) Gold 6338 CPUs using 20 CPU cores. The experiments involved varying the number of reference sources (e.g., 15, 45, 75) as input for both tools. Each source contains about 2,000 to 10,000 taxa (after removing taxa with zero abundance in both X and Y). We recorded the running time and memory usage of SourceID-NMF and FEAST, as illustrated in Figure S1.

Both tools exhibited an increasing running time as the number of reference sources increased. With a small number of sources (e.g., 15), both SourceID-NMF and FEAST were capable of completing the estimation within an hour. However, when using a larger number of sources (i.e., 75), SourceID-NMF took several hours to complete the estimation, while FEAST finished in approximately an hour. Thus, FEAST demonstrated a significant advantage in terms of running time compared to SourceID-NMF. The longer running time of SourceID-NMF can be attributed to the computation of matrix inversion related to the number of input sources. Thus, reducing the number of input sources will speed up the process. One possible approach is to cluster the sources and use representative sources as input. We have incorporated this option into our tool for the convenience of our users. Additionally, we have implemented a multi-threaded process to enhance the speed of SourceID-NMF, enabling users to leverage multiple CPU cores for faster execution. And we are actively exploring the use of other faster optimization algorithms for SourceID-NMF in future iterations. Although SourceID-NMF is more time-consuming, it requires less memory compared to FEAST. The low memory usage makes SourceID-NMF suitable for running on general devices.

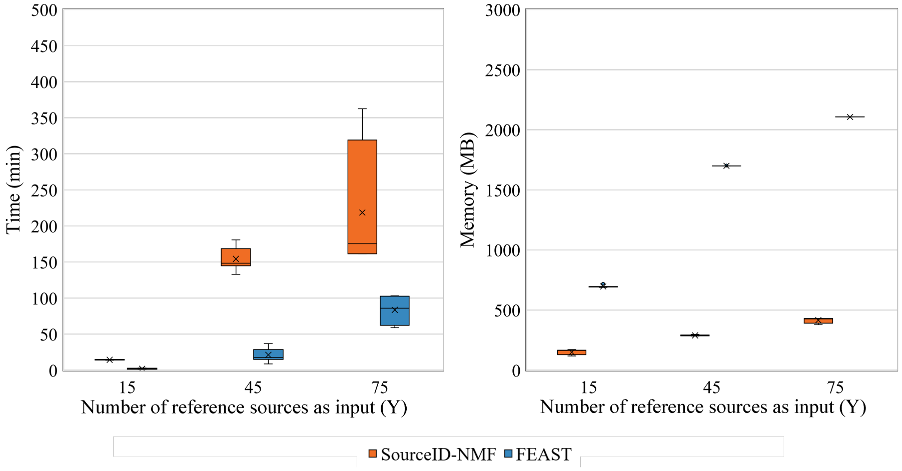

Figure S1: The running time and memory usage of SourceID-NMF and FEAST on datasets with various number of reference sources as input. X-axis: the number of reference sources. Y-axis: the running time and maximum memory usages, respectively.

3 Similarity between W and true sources analysis

In the optimization objective of SourceID-NMF we estimate both H and W. In the main draft, we compared the estimated H with the true source proportions. In this section, we add metrics to check the similarity between the estimated W and the true sources. Ideally, they should have observable similarity but not necessarily identical. Based on the original dataset with different inter-source divergences (0.6, 0.7, and 0.8 JSD), we compute the W generated by each set of sources for each target sample.We then compare W with the abundance of true sources, which measures the degree of similarity between the estimated source's and the corresponding true sources by JSD. For unknown sources, the W of the true unknown source is an integration of the 5 unknown sources with their corresponding proportions. It can be calculated using the formula $W_u = \sum_j^5 W_j * H_j / \sum_j^5 H_j$. In Fig. 2, the x-axis represents three different sets of inter-source divergences (0.6, 0.7, and 0.8 JSD), and the y-axis represents the JSD between the corresponding sources of W and true sources.

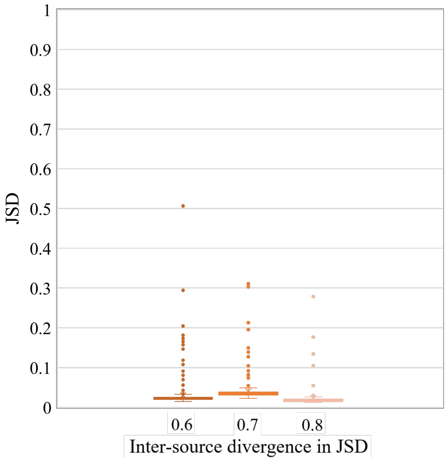

Figure S2: Similarity between estimated W and true sources using JSD. X-axis: averaged pairwise divergence (0.6, 0.7, and 0.8 JSD) between 20 sources, measured by JSD on taxa abundance. Y-axis: JSD between the estimated W and the abundance of true sources

As shown in Fig. S2, SourceID-NMF's estimation of W is close to that of true sources. The JSD between W and true sources remains between 0-0.1 for most known sources. Only a few unknown sources have some offset from the true results, which is between 0.1 and 0.3, but does not affect the final H estimation. This is also due to the fact that SourceID-NMF is accurate for most sources in W so as to ensure the accuracy of the estimated proportions.

4 Impact of varying the number of irrelevant sources on performance

In this section, we investigated the impact of a different number of irrelevant sources on SourceID-NMF. We used the datasets with inter-source divergence of 0.6, 0.7, and 0.8 JSD from the experiments in Section 3.1.2 of the main manuscript. Each dataset contained 5 irrelevant sources. By varying the number of irrelevant sources (1, 3, 5), we applied SourceID-NMF to estimate the

source proportions for the target samples. Figure S3 shows the JSD values between the estimated proportions and the ground-truth proportions, as well as the boxplot of estimated proportions for the irrelevant sources. It is reasonable that SourceID-NMF estimated more irrelevant sources as potential sources when more irrelevant sources were used as input. However, most of the estimated proportions for irrelevant sources were extremely low, close to zero. As a result, the estimated proportions of other sources were not significantly affected, as shown in Figure S3 (a). Since irrelevant sources are typically different from the actual sources mixed in the target samples, using different numbers of irrelevant sources did not affect the estimation results of SourceID-NMF.

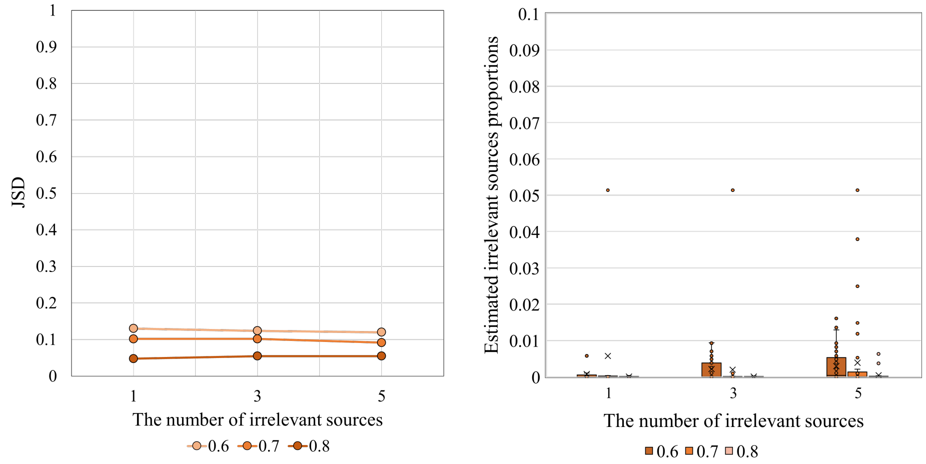

(a) (b)

Figure S3: The performance of applying SourceID-NMF to target samples with inter-source divergence of 0.6, 0.7 and 0.8 JSD from the experiments in Section 3.1.2 of the main manuscript, using various number of irrelevant sources. (a) The JSD values between the estimated sources proportions and the ground-truth proportions. (b) Boxplot of the estimated proportions of irrelevant sources.

5 Further analysis on tracking potential sources in infants' fecal samples

In the analysis of infant fecal samples, SourceID-NMF exclusively identified the tube as a known source for the fecal samples. In contrast, FEAST returns more sources including hands, environmental surfaces, and incubators. In this section, we conducted a systematic experiment to thoroughly investigate the reasons behind the different outputs.

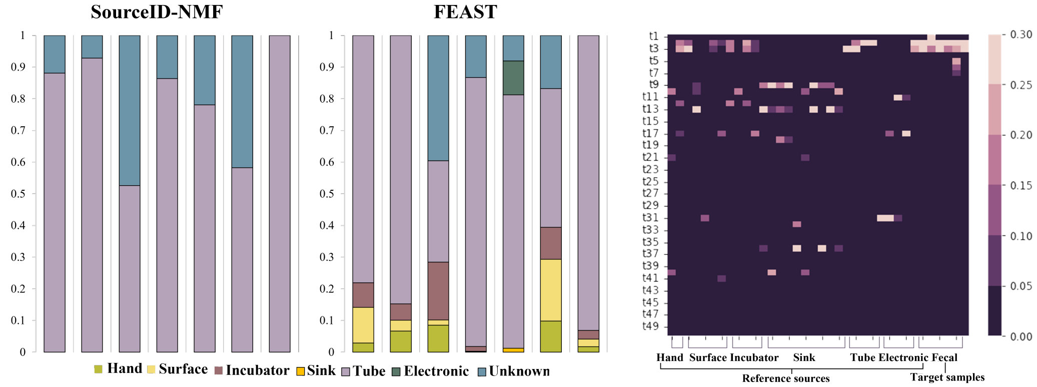

(a) (b)

Figure S4: The results of SourceID-NMF and FEAST in tracking potential sources of gut-colonizing microbes in infants from NICUs. (a) The estimated source proportions in the fecal sample. (b) The heat map represents the relative taxa abundance in sources and fecal samples. The rows correspond to the 50 most abundant taxa across different samples, while the columns represent the sources and fecal samples. (This is a duplication of Fig. 10 in the main draft.)

As Figure S4 shows, the fecal samples' major taxonomic groups are much fewer compared to many sources. The additional sources returned by FEAST have many unique taxa that are not present in the target sample. Therefore, SourceID-NMF did not consider them as potential contributors. Based on the taxa composition as shown in the heatmap, we constructed simulated data to mimic that data characteristics. We make a special hard case by directly dropping taxa in the target sample so that more sources can contain unique taxa w.r.t. the target sample. The simulated data construction contains two steps. First, we created three target samples using the 29 sources. For each target sample, the proportion of every source is determined using the contributions

of sources to the corresponding target sample estimated by FEAST. The relative contribution of each source in the three target samples are shown in Figure S6. In the simulation, we used the four electronic samples as unknown sources to create the target samples because they were not detected by the two tools in the real data experiment. Therefore, we had three target samples and 25 reference samples (Y) from hand, surface, incubator, sink, and tube. Next, we started to remove taxa in the target sample so that we have a similar case as the real data experiment. By ranking all taxa in descending order based on their abundance, we retain the top 100%, 80%, 50%, 20%, 15%, and 10% of taxa, respectively. Thus, we created a total of 3×6 target samples with different levels of missing taxa. We applied SourceID-NMF and FEAST to these target samples and summarized their results in Figure S6.

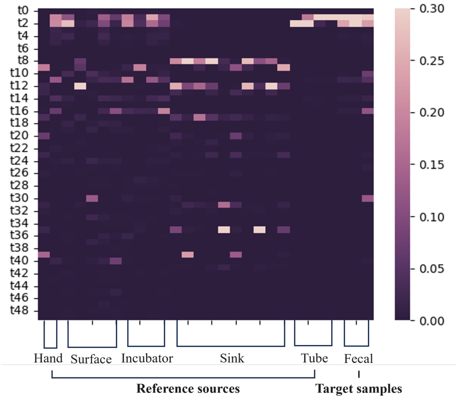

Figure S5: The heat map represents the relative taxa abundance in reference sources and three simulated target samples with 20% retained taxa. The rows correspond to the 50 most abundant taxa across different samples, while the columns represent the sources and target sample.

When the retained portion of taxa in the target samples is high (e.g., >50%), both SourceID-NMF and FEAST detected all the associated sources. When the target samples only keep the top 20% dominant taxa, SourceID-NMF started to regard some sources as irrelevant because the reference sources have significant number of unique taxa as shown in Figure S5. This observation aligns with the findings from the real data experiments. Interestingly, we discovered that even though our tools failed to identify the low-proportion sources when the target samples were missing many taxa, the estimated proportions of the dominant source and the unknown sources remained close to the ground truth. In particular, SourceID-NMF exhibited higher accuracy in estimating the unknown sources, whereas FEAST underestimated the proportion of the unknown source while overestimating the low-proportion sources significantly.

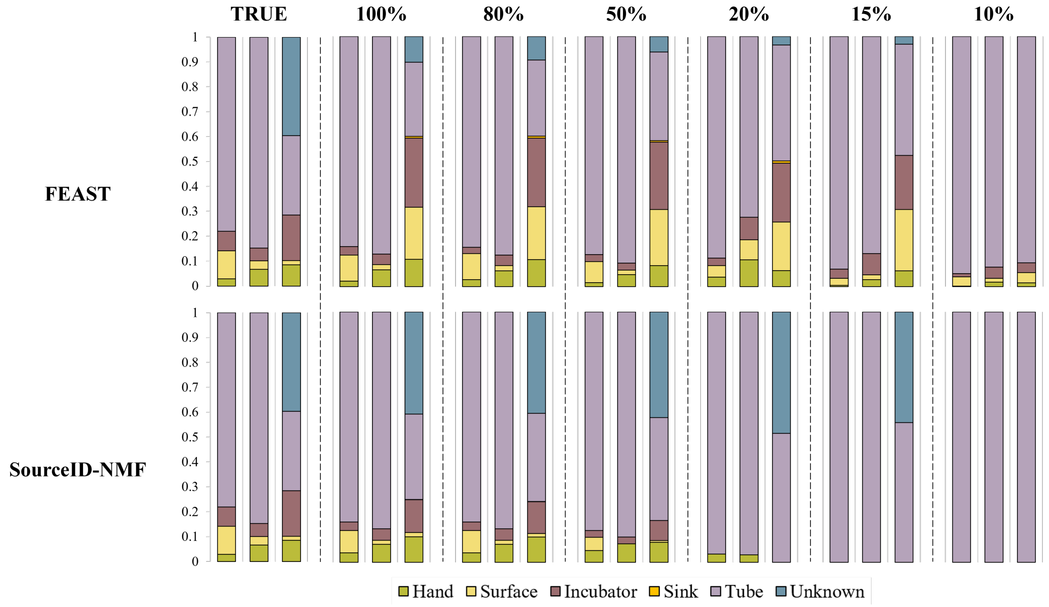

Figure S6: The estimated proportions of FEAST and SourceID-NMF on target samples with different levels of retained taxa, e.g., 80% means we keep 80% dominant taxa with filtering 20% low-abundance taxa (set their abundance as zero) in the original target samples.

Considering the need of users who prefer a more aggressive source tracking, we provide an option for SourceID-NMF to include more sources in the prediction. When there are many observed taxa in the reference sources but much fewer ones in the target samples, we consider keeping only the taxa that are present in the target samples for all the reference sources and then

conduct source tracking using SourceID-NMF. Figure S7 (a) illustrates the estimated source proportions (denoted by 'OPTION') obtained by applying SourceID-NMF to the target samples, with the reference samples only retaining the taxa presented in the target samples. We observed that after removing unique taxa from the reference (source) samples, SourceID-NMF can return more sources. This demonstrates the feasibility of the strategy for enabling SourceID-NMF to detect more sources in this special scenario. Additionally, we found that the estimated proportion of dominant sources and the unknown source maintained good accuracy. Although this strategy may introduce irrelevant sources, the estimated proportions for the irrelevant sources were low. Thus, we utilized this strategy to analyze the real fecal data and reported the estimated proportions of sources in Figure S7 (b). We found that SourceID-NMF detected more low-proportion sources, similar to FEAST in Fig. 10 (a) (main manuscript). Furthermore, the proportions of dominant sources and unknown sources were not significantly affected, which is consistent with the results from the above simulated experiments. In conclusion, we provide an optional strategy for SourceID-NMF to conduct a more aggressive detection of sources.

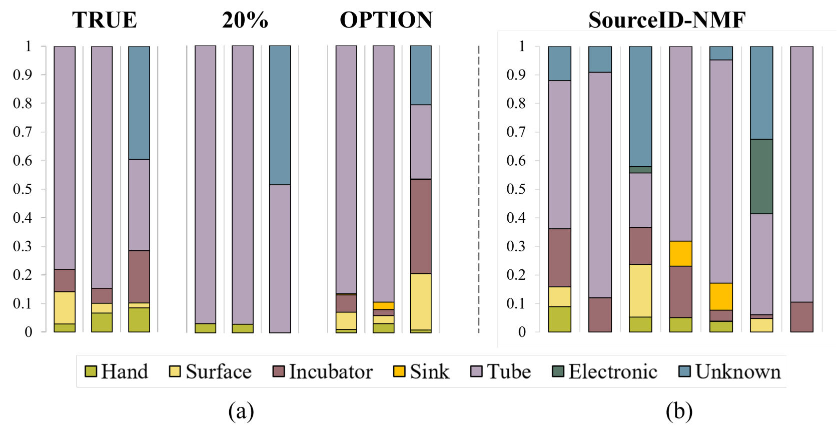

Figure S7: The estimated proportions of SourceID-NMF using the strategy that reference sources only keep the observed taxa in the target sample. (a) The new strategy (denoted as 'OPTION') on the same target sample with 20% retained taxa. (b) The estimated proportions of sources on the real fecal samples using the new strategy.

As we speculate that FEAST tends to overly estimate the contribution of sources that share taxa with the target samples. To examine this, we conducted another experiment. We used a similar approach to generate three target samples with the estimated proportions on the first three fecal samples by SourceID-NMF shown in Figure S8. In this simulation, only samples from tube and the unknown source (electronic) have non-zero proportions. Thus, irrelevant samples from other sources contains unique taxa compared with the target samples. We then applied SourceID-NMF and FEAST to these target samples and summarized their results in Figure S8. From Figure S8, we observed that FEAST estimated a significant number of irrelevant sources, including hands, surfaces, and incubators. It seems that FEAST tends to overly include more sources if they share some taxa with the target samples, leading to the inclusion of more irrelevant sources and underestimating the proportion of unknown sources. This observation aligns with the results from the simulated experiments. In comparison, SourceID-NMF performs well in estimating the proportions of unknown sources and estimating fewer irrelevant sources with non-zero proportions.

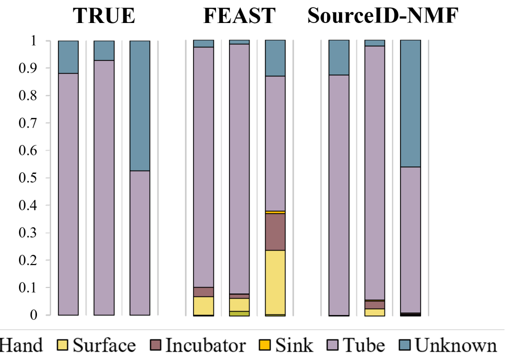

Figure S8: The estimated proportions of sources by FEAST and SourceID-NMF on three simulated target samples, with specific sources contributions shown in 'TRUE'.

In conclusion, when analyzing infant's fecal samples in real data experiments, SourceID-NMF and FEAST exhibit similar estimations for dominant sources but display different biases when estimating low-proportion sources. This bias arises due to

the unique taxa abundance profiles in the data, whereby the reference sources share some observed taxa with the target samples but also contain many unique taxa that are unobserved in the target samples. This property could be attributed to missing taxa in the target samples due to sequencing errors, or it is possible that sources with many unique taxa are irrelevant sources. To investigate these two cases, we conducted simulated experiments. The overall performance in estimating the proportions of sources for all experiments is visualized in Figure S9. In the first case, when the target samples missed a significant number of certain taxa, SourceID-NMF tended to regard them as irrelevant. To make SourceID-NMF more aggressive, we proposed a strategy for identifying low-proportion sources by accounting for the missing taxa in the target samples. In the second case, FEAST overestimated the proportions of irrelevant sources, while SourceID-NMF yielded fewer irrelevant sources in the results, mitigating this overestimation. These two experiments, along with the real data experiment, provide evidence that FEAST tends to include more potential sources, resulting in a higher recall but also a higher false positive rate. Additionally, FEAST underestimates the proportions of unknown sources. On the other hand, SourceID-NMF demonstrates higher precision in identifying potential sources and provides more accurate estimations of the proportions of unknown sources. However, SourceID-NMF may miss some low-proportion sources in the process. To uncover more potential sources for SourceID-NMF, we have proposed a possible strategy to address the case.

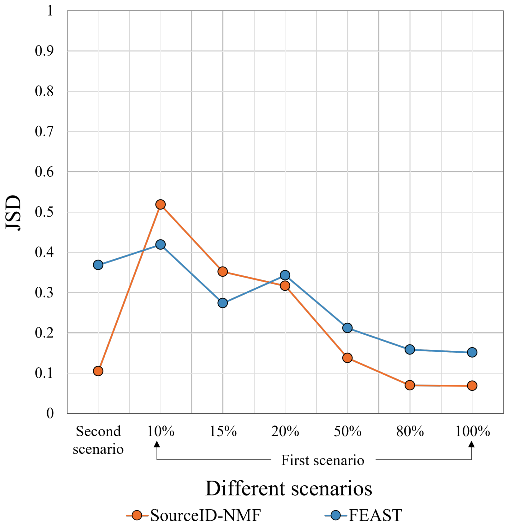

Figure S9: The average JSD values between the estimated proportions of sources and the ground-truth proportions for SourceID-NMF and FEAST on target samples generated from two scenarios.

6 Other Supplementary Figures

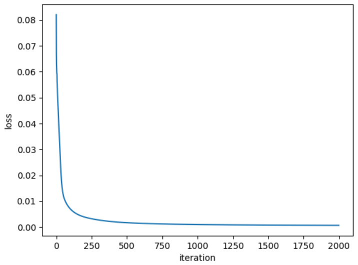

(a) A simulated sample with 0.6 JSD

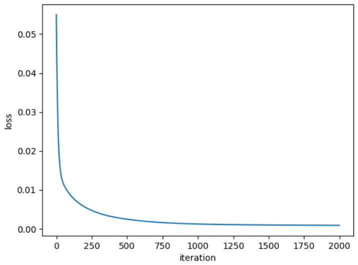

(b) A simulated sample with 0.7 JSD

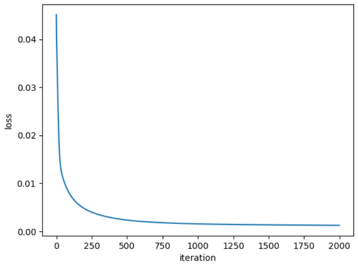

(c) A simulated sample with 0.8 JSD

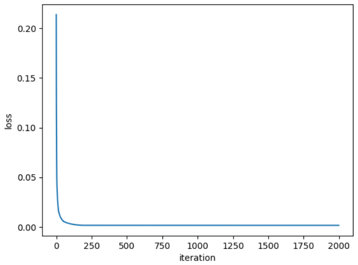

(d) The third fecal sample

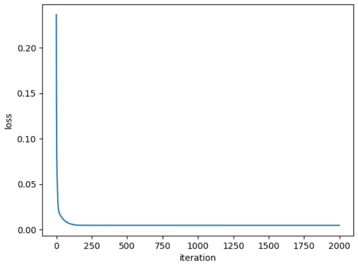

(e) The fourth fecal sample

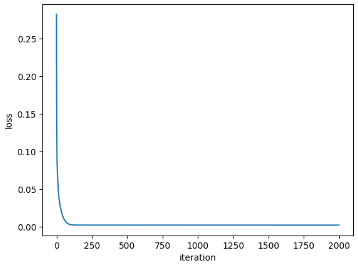

(f) The fifth fecal sample

Figure S10: The evolution of the loss function for three simulated target samples with inter-source divergences of 0.6, 0.7, and 0.8 JSD, based on the experiments conducted in Section 3.1.2 of the main manuscript, and three fecal samples from the second real data experiment.

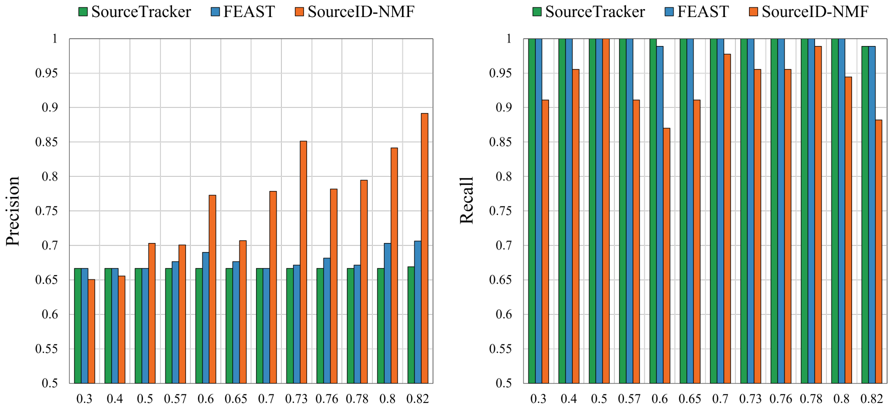

Figure S11: Performance of three tools on simulated datasets with varying inter-source divergence. X-axis: averaged pairwise divergence between 20 sources, measured by JSD on taxa abundance. Left to right: increased inter-source divergence. Y-axis in two plots: Precision and Recall value between the estimated and the true source proportions. A threshold of 1E-06 was set to determine source identification.

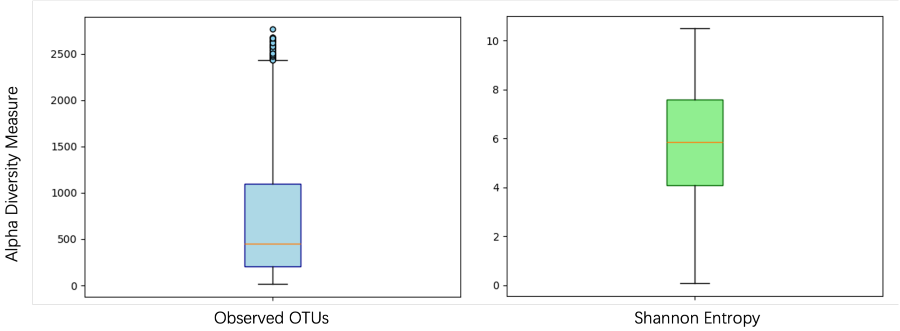

Figure S12: The alpha diversity measures of the abundance profiling table from the Earth's Microbiome project using the "Observed OTUs" and "Shannon Entropy" metrics.

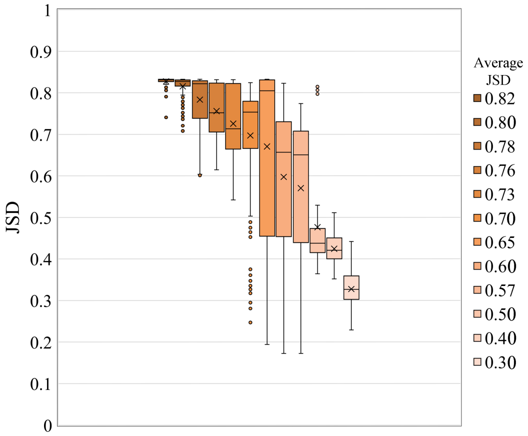

Figure S13: The pair-wise inter-source divergences in 12 sets of sources (each contains 20 sources) used for generating the simulated data.

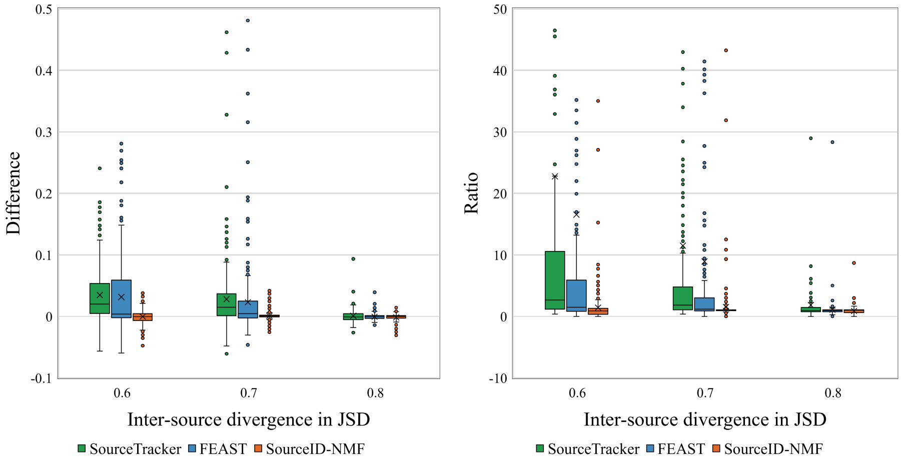

Figure S14: The boxplots of the estimated proportions for the low abundance sources (below 0.1 proportions) by three tools on the simulated data with the inter-source divergence in JSD of 0.8, 0.7, and 0.6. X-axis: averaged pairwise divergence between 20 sources. Y-axis in two plots: Difference and Ratio (estimation divided by the ground-truth) between estimated proportions and true proportions.

References

[1] Stephen Boyd, Neal Parikh, Eric Chu, Borja Peleato, Jonathan Eckstein, et al. Distributed optimization and statistical learning via the alternating direction method of multipliers. *Foundations and Trends® in Machine learning*, 3(1):1–122, 2011.

[2] Dennis L Sun and Cedric Fevotte. Alternating direction method of multipliers for non-negative matrix factorization with the beta-divergence. In *2014 IEEE international conference on acoustics, speech and signal processing (ICASSP)*, pages 6201–6205. IEEE, 2014.

[3] Yangyang Xu, Wotao Yin, Zaiwen Wen, and Yin Zhang. An alternating direction algorithm for matrix completion with nonnegative factors. *Frontiers of Mathematics in China*, 7:365–384, 2012.

[4] Yin Zhang. An alternating direction algorithm for nonnegative matrix factorization. *preprint*, 2010.

[5] Stephen P Boyd and Lieven Vandenberghe. *Convex optimization*. Cambridge University Press, 2004.

[6] Pauli Virtanen, Ralf Gommers, Travis E Oliphant, Matt Haberland, Tyler Reddy, David Cournapeau, Evgeni Burovski, Pearu Peterson, Warren Weckesser, Jonathan Bright, et al. Scipy 1.0: fundamental algorithms for scientific computing in python. *Nature methods*, 17(3):261–272, 2020.
